# Li–Yau inequalities for the Helfrich functional and applications

**DOI:** 10.1007/s00526-022-02381-7

**Published:** 2022-12-24

**Authors:** Fabian Rupp, Christian Scharrer

**Affiliations:** 1 Faculty of Mathematics, University of Vienna, Oskar-Morgenstern-Platz 1, 1090 Vienna, Austria; 2 Institute for Applied Mathematics, University of Bonn, Endenicher Allee 60, 53115 Bonn, Germany

**Keywords:** 53C42 (primary), 49Q10, 92C10 (secondary)

## Abstract

We prove a general Li–Yau inequality for the Helfrich functional where the spontaneous curvature enters with a singular volume type integral. In the physically relevant cases, this term can be converted into an explicit energy threshold that guarantees embeddedness. We then apply our result to the spherical case of the variational Canham–Helfrich model. If the infimum energy is not too large, we show existence of smoothly embedded minimizers. Previously, existence of minimizers was only known in the classes of immersed bubble trees or curvature varifolds.

## Introduction

*Lipid bilayers* make up the cellular membranes of most organisms. These extremely thin structures commonly form vesicles, so mathematically they are naturally modeled as two-dimensional structures, i.e. closed surfaces. The *Canham–Helfrich model* [10, 19] characterizes the equilibrium shapes of lipid bilayers as (constrained) minimizers of a curvature dependent bending energy. For an oriented surface $\Sigma$ and an immersion $f:\Sigma \rightarrow {\mathbb {R}}^3$, the *Helfrich energy* is defined by

$$\begin{aligned} {\mathcal {H}}_{c_0}(f) :=\frac{1}{4}\int \limits _{\Sigma }|H-c_0n|^2\mathop {}\!\textrm{d}\mu . \end{aligned}$$

Here, $H=H_f$ is the mean curvature vector of the immersion, i.e. the trace of the second fundamental form, $n=n_f$ is the unit normal induced by the orientation of $\Sigma$ (see (2.6) below) and $\mu =\mu _f$ denotes the Riemannian measure associated to the pullback metric $g=g_f=f^{*}\langle \cdot ,\cdot \rangle$ on $\Sigma$, where $\langle \cdot ,\cdot \rangle$ denotes the Euclidean inner product in ${\mathbb {R}}^3$. The constant $c_0\in {\mathbb {R}}$ is called *spontaneous curvature*. Since *H* is normal to the surface, the Helfrich energy can also be written as

$$\begin{aligned} {\mathcal {H}}_{c_0}(f) =\frac{1}{4}\int \limits _{\Sigma }(H_{\textrm{sc}}-c_0)^2\mathop {}\!\textrm{d}\mu , \end{aligned}$$

where $H_{\textrm{sc}}:=\langle H, n\rangle$ is the scalar mean curvature with respect to *n*. We choose the inner unit normal such that the standard embedding of the round sphere of radius $r>0$ has positive scalar mean curvature $H_{\textrm{sc}} = \frac{2}{r} >0$. In particular, if $c_0>0$, the Helfrich energy is zero for a round sphere of appropriately chosen radius. Reversing the orientation on $\Sigma$ corresponds precisely to replacing *n* by $-n$. Thus, we have

1.1 $$\begin{aligned} {\mathcal {H}}_{c_0}(f) = {\mathcal {H}}_{-c_0}({\hat{f}}), \end{aligned}$$

where ${\hat{\Sigma }}$ is the surface $\Sigma$ with reversed orientation and ${\hat{f}}:{\hat{\Sigma }}\rightarrow {\mathbb {R}}^3, {\hat{f}}(p)=f(p)$. Clearly, the Helfrich functional is not scale-invariant. However, we observe the following scaling property involving both arguments:

$$\begin{aligned} {\mathcal {H}}_{c_0}(f) = {\mathcal {H}}_{\frac{c_0}{r}}(rf)\qquad \text {for }r>0. \end{aligned}$$

In particular, we see that

1.2 $$\begin{aligned} \lim _{r\rightarrow 0+}{\mathcal {H}}_{c_0}(rf) = \lim _{r\rightarrow 0+}{\mathcal {H}}_{rc_0}(f) = {\mathcal {H}}_{0}(f) =:{\mathcal {W}}(f). \end{aligned}$$

The right hand side is well known as the *Willmore energy*. In contrast to the Helfrich functional, the Willmore functional ${\mathcal {W}}$ is scale-invariant and does not depend on the unit normal field *n* or the orientation of the underlying surface $\Sigma$. One may also consider the $L^2$-CMC-deficit

1.3 $$\begin{aligned} {\bar{{\mathcal {H}}}}(f):=\inf _{c_0\in {\mathbb {R}}}{\mathcal {H}}_{c_0}(f) = \frac{1}{4}\int \limits _\Sigma (H_{\textrm{sc}} - \bar{H}_{\textrm{sc}})^2\mathop {}\!\textrm{d}\mu , \end{aligned}$$

where  is the average scalar mean curvature. Also the functional ${\bar{{\mathcal {H}}}}$ is scale-invariant and does not depend on the orientation of $\Sigma$. For more details and corresponding results, see Sects. 5.2 and 6.4.

We are primarily interested in the following minimization problem suggested by Canham [10] and Helfrich [19] in order to study the shape of red blood cells. Our main contribution is stated in Theorem 1.6 below (see also Theorem 6.10).

### Problem 1.1

Let $c_0\in {\mathbb {R}}$ and $A_0,V_0>0$ be given constants. Let the unit sphere ${\mathbb {S}}^2\subset {\mathbb {R}}^3$ be oriented by the inner unit normal. Minimize the functional ${\mathcal {H}}_{c_0}$ in the class of smooth embeddings $f:{\mathbb {S}}^2\rightarrow {\mathbb {R}}^3$ subject to the constraints

$$\begin{aligned} {\mathcal {A}}(f):=\int \limits _{{\mathbb {S}}^2} 1\mathop {}\!\textrm{d}\mu = A_0,\qquad {{\,\mathrm{{\mathcal {V}}}\,}}(f):=-\frac{1}{3}\int \limits _{{\mathbb {S}}^2}\langle f,n\rangle \mathop {}\!\textrm{d}\mu = V_0. \end{aligned}$$

We consider the following example of Problem 1.1 where the infimum cannot be attained by a smooth embedding, cf. [31].

### Example 1.2

Let $\iota _{{\mathbb {S}}^2}:{\mathbb {S}}^2\rightarrow {\mathbb {R}}^3$ be the inclusion of the unit sphere. Let $c_0 :=2$, $A_0 :=2{\mathcal {A}}(\iota _{{\mathbb {S}}^2})$, and $V_0:=2{{\,\mathrm{{\mathcal {V}}}\,}}(\iota _{{\mathbb {S}}^2})$. There exists a sequence of smooth embeddings $f_k:{\mathbb {S}}^2\rightarrow {\mathbb {R}}^3$ satisfying ${\mathcal {A}}(f_k) = A_0$ and ${{\,\mathrm{{\mathcal {V}}}\,}}(f_k) = V_0$ which converges in the varifold topology to the set $T\subset {\mathbb {R}}^3$ given by two translations of the unit sphere that meet in exactly one point (see Fig. 2a) such that

1.4 $$\begin{aligned} \lim _{k\rightarrow \infty }{\mathcal {H}}_{c_0}(f_k) = 2{\mathcal {H}}_{c_0}(\iota _{{\mathbb {S}}^2}) = 0. \end{aligned}$$

**Fig. 1:**
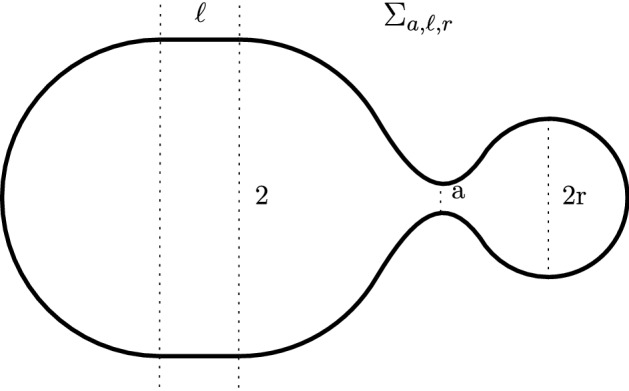
Visualization of the construction

To see this, let $\Sigma _{\ell ,r}$ be the spherical $C^{1,1}$-regular surface that results by gluing two spherical caps at the ends of a cylinder of length $\ell \ge 0$ with radius $r>0$. Denote with $\Sigma _{0,\ell ,r}$ the disjoint union of $\Sigma _{\ell ,1}$ with $\Sigma _{0,r}$ (a sphere with radius *r*) and with $\Sigma _{a,\ell ,r}$ the spherical $C^{1,1}$-regular surface that results by connecting $\Sigma _{\ell ,1}$ with $\Sigma _{0,r}$ through a catenoidal bridge of small neck size $a>0$, cf. Fig. 1. The sequence $(\Sigma _{k^{-1},0,1})_{k\in {\mathbb {N}}}$ satisfies (1.4). However, the gluing only gives $C^{1,1}$-regularity and the conditions on area and volume are not met. We will first adjust the isoperimetric ratio defined by ${\mathcal {I}} :={\mathcal {A}}^3/{\mathcal {V}}^2$. A short computation reveals that $I_{a,\ell ,r}:=\mathcal I(\Sigma _{a,\ell ,r})$ satisfies

$$\begin{aligned} I_{0,\ell ,1}> I_{0,0,1} =:I_0 = A_0^3/V_0^{2}> I_{0,0,r}\qquad \text { for } \, \ell >0 \, \text { and } \, 0<r<1. \end{aligned}$$

Moreover, $I_{a,\ell ,r}$ depends continuously on $a\ge 0$, $\ell \ge 0$ and $r>0$. Hence, if $k\in {\mathbb {N}}$ and $I_{k^{-1},0,1} > I_0$, we can first choose $1-k^{-1}<r_k<1$ such that still $I_{k^{-1},0,r_k} > I_0$ and then $0<a_k<k^{-1}$ with $I_{a_k,\ell _k,r_k} = I_0$ where $\ell _k = 0$. If on the other hand $k\in {\mathbb {N}}$ and $I_{k^{-1},0,1} < I_0$, we can first choose $0<\ell _k<k^{-1}$ such that still $I_{k^{-1},\ell _k,1} < I_0$ and then $0<a_k < k^{-1}$ with $I_{a_k,\ell _k,r_k} = I_0$ where $r_k = 1$. Now, we let $S_k = \Sigma _{a_k,\ell _k,r_k}$ and choose $\lambda _k>0$ such that ${\mathcal {A}}(\lambda _k S_k) = A_0$ and ${{\,\mathrm{{\mathcal {V}}}\,}}(\lambda _k S_k) = V_0$. Then, since $a_k\rightarrow 0$, $\ell _k\rightarrow 0$, and $r_k\rightarrow 1$ as $k\rightarrow \infty$, there holds ${\mathcal {A}}(S_k)\rightarrow A_0$, so ${{\,\mathrm{{\mathcal {V}}}\,}}(S_k)\rightarrow V_0$ and $\lambda _k\rightarrow 1$. It follows that also the sequence $(\lambda _kS_k)_{k\in {\mathbb {N}}}$ satisfies (1.4).

In order to have $C^\infty$-regularity, one can apply mollifications near the patching regions and subsequently compensate possible changes of area and volume using [32, Lemma 2.1] supported on the catenoid away from the mollifiers.

Consequently, the infimum of Problem 1.1 is attained by the set *T*. Clearly, *T* cannot be written as the image of a smooth immersion of ${\mathbb {S}}^2$. If on the other hand $f:{\mathbb {S}}^2\rightarrow {\mathbb {R}}^3$ is a smooth immersion with ${\mathcal {H}}_{c_0}(f) = 0$ then, by a result of Hopf [20, Chapter VI, Theorem 2.1], the image of *f* must be a round sphere. In particular, if $f:{\mathbb {S}}^2 \rightarrow {\mathbb {R}}^3$ is a smooth embedding with ${\mathcal {H}}_{c_0}(f) = 0$, then ${\mathcal {A}}(f) \ne A_0$ and ${{\,\mathrm{{\mathcal {V}}}\,}}(f)\ne V_0$.

In the terminology of [30, 31], the set *T* in Example 1.2 is the *bubble tree* consisting of two unit spheres. Bubbling phenomena have also been observed in nature and are known as *budding transition* [42]. Thus, the space of bubble trees appears to be a natural class in which to minimize the Helfrich functional. Indeed, in [31, Theorem 1.7], the existence of minimizers for the Helfrich functional in the class of immersed bubble trees was proven. Each of the bubbles is given by a map ${\mathbb {S}}^2\rightarrow {\mathbb {R}}^3$ which outside of finitely many so called *branch points* is a smooth immersion. For similar results, see [11, 15]. However, not all minimizers of Problem 1.1 are necessarily bubble trees, consider for instance the case $c_0=2$, $A_0={\mathcal {A}}(\iota _{{\mathbb {S}}^2})$, $V_0 = {{\,\mathrm{{\mathcal {V}}}\,}}(\iota _{{\mathbb {S}}^2})$. One may conjecture that bubbling can only occur if the parameters $A_0$ and $V_0$ are within a certain range depending on $c_0$. Apart from the geometric relevance to obtain such qualitative results for the minimizers of Problem 1.1, it is of great interest to confirm mathematically that the Canham–Helfrich model is suitable for the study of red blood cells which are actually embedded—rather than a bubble tree. As a first step in this direction it was proven in [31] that there exists a constant $\varepsilon = \varepsilon (A_0,V_0)>0$ such that the minimizers are given by smooth embeddings provided $|c_0|<\varepsilon$. However, apart from the fact that $\varepsilon (A_0,V_0)$ is implicitly small, one would rather want to have a criterion of the following type: *For all* $c_0\in {\mathbb {R}}$, *the Problem* 1.1*has a solution provided* $A_0$ *and* $V_0$ *are in a certain explicit range depending on* $c_0$.

The proof of embeddedness of minimizers in [31] is based on the fact that for $|c_0|$ small, the Helfrich functional is close to the Willmore functional, see (1.2), and minimizers for $c_0=0$ are given by smooth embeddings, see [41]. A crucial tool to prove smoothness and embeddedness of the minimizers in [41] (i.e. solutions of Problem 1.1 for $c_0=0$) is the following inequality of Li and Yau [25, Theorem 6]. If $\Sigma$ is *closed* (i.e. compact and without boundary), for any $x_0\in {\mathbb {R}}^3$ we have

1.5 $$\begin{aligned} {\mathcal {H}}^{0}(f^{-1}\{x_0\}) \le \frac{1}{4\pi }{\mathcal {W}}(f), \end{aligned}$$

where ${\mathcal {H}}^{0}$ denotes the counting measure. In particular, if ${\mathcal {W}}(f)<8\pi$, then *f* must be an embedding. This observation also played an essential role in the study of the Willmore energy and related topics, cf. for instance [22–24, 33, 35, 41, 43].

In view of the fact that branch points have multiplicity at least 2, such a tool could be the key to exclude bubbling in the Canham–Helfrich model. Apart from comparing the Helfrich energy with the Willmore energy for small $|c_0|$ via (1.2), one might also try to make use of the Li–Yau inequality (1.5) by estimating the Willmore energy from above in terms of the Helfrich energy (see (2.5)):

1.6 $$\begin{aligned} {\mathcal {W}}(f) \le 2{\mathcal {H}}_{c_0}(f) + \frac{1}{2}c_0^2{\mathcal {A}}(f). \end{aligned}$$

Again, if the right hand side is strictly less than $8\pi$, then the Li–Yau inequality (1.5) implies that *f* is an embedding. However, as one of our results reveals (see Lemma 6.1), in the case of red blood cells where $c_0<0$ (see [13]), there holds ${\mathcal {H}}_{c_0}(f)>4\pi$. In particular, the right hand side of (1.6) is already strictly larger than $8\pi$ and one cannot apply the Li–Yau inequality (1.5) to deduce embeddedness of *f*.

Another naive attempt to apply (1.5) would be to show that ${\mathcal {W}}\le {\mathcal {H}}_{c_0}$ for $c_0<0$. However, this is impossible by the following simple scaling argument. Let $f:{\mathbb {S}}^2\rightarrow {\mathbb {R}}^3$ be an immersion such that $\int _{{\mathbb {S}}^2}H_{\textrm{sc}}\mathop {}\!\textrm{d}\mu <0$ (such an *f* exists by [12, Theorem 1.2]). We find that

$$\begin{aligned} {\mathcal {H}}_{c_0}(rf)-{\mathcal {W}}(rf) = -\frac{rc_0}{2}\int \limits _{{\mathbb {S}}^2} H_{\textrm{sc}}\mathop {}\!\textrm{d}\mu + \frac{r^2 c_0^2}{4}~{\mathcal {A}}(f) \end{aligned}$$

which becomes negative if $r>0$ is sufficiently small as $c_0<0$.

### Main results

Instead of applying (1.5) by comparing the Helfrich energy with the Willmore energy, the aim of this article is to prove and apply a Li–Yau type inequality directly for the Helfrich functional. In the smooth setting, our multiplicity inequality reads as follows.

#### Lemma 1.3

Let $f:\Sigma \rightarrow {\mathbb {R}}^3$ be a smooth proper immersion of an oriented surface $\Sigma$ without boundary. Let $c_0\in {\mathbb {R}}$, $x_0\in {\mathbb {R}}^3$, and suppose that the *concentrated volume of* *f* *at* $x_0$ defined by

1.7 $$\begin{aligned} {{\,\mathrm{{\mathcal {V}}}\,}}_{c}(f, x_0) :=- \int \limits _{\Sigma }\frac{\langle f-x_0, n\rangle }{|f-x_0|^2}\mathop {}\!\textrm{d}\mu \end{aligned}$$

exists. Then

1.8 $$\begin{aligned} {\mathcal {H}}^0(f^{-1}\{x_0\}) \le \limsup _{\rho \rightarrow \infty } \frac{\mu (f^{-1}(B_\rho (x_0)))}{\pi \rho ^2}+ \frac{1}{4\pi } {\mathcal {H}}_{c_0}(f) + \frac{c_0}{2\pi }{{\,\mathrm{{\mathcal {V}}}\,}}_c(f,x_0). \end{aligned}$$

In order to apply Eq. (1.8), it is of crucial interest to determine the sign of the concentrated volume. Despite singular, the integrand in (1.7) is subcritical and locally integrable, see Lemma 3.2 and Remark 3.3 below. Moreover, the integrand is nonpositive if $f[\Sigma ]$ parametrizes the boundary of an open set in ${\mathbb {R}}^3$ which is star-shaped with respect to $x_0$ and *n* is the inner unit normal, cf. [16, 9.4.2]. However, such an immersion *f* must be embedded a priori.

It turns out that the sign of the concentrated volume can be determined if we can find a suitable notion of inner unit normal, resulting in an appropriate divergence theorem.

#### Definition 1.4

We call a smooth immersion $f:\Sigma \rightarrow {\mathbb {R}}^3$ of a closed surface $\Sigma$ an *Alexandrov immersion*, cf. [1], if there exist a smooth compact 3-manifold *M* with boundary $\partial M=\Sigma$, a smooth inner unit normal field $\nu$ to $\Sigma$ and a smooth immersion $F:M\rightarrow {\mathbb {R}}^3$ such that $F\vert _{\Sigma }=f$. The surface $\Sigma$ is then necessarily orientable. Moreover, we choose the orientation on $\Sigma$ such that the induced normal field along *f* (see (2.6) below) satisfies $n=\mathop {}\!\textrm{d}F(\nu )$.

Our orientation on $\Sigma$ does not coincide with the usual Stokes orientation. The reason for this is that we want to work with the inner unit normal such that the standard embedding of a round sphere has positive scalar mean curvature.

In the setting of Definition 1.4, the Li–Yau inequality (1.8) can be put into the following more convenient form.

#### Theorem 1.5

Let $\Sigma$ be a closed surface and let $f:\Sigma \rightarrow {\mathbb {R}}^3$ be an Alexandrov immersion with $f=F\vert _{\Sigma }$, $F:M\rightarrow {\mathbb {R}}^3$ as in Definition 1.4. Then for all $x_0\in {\mathbb {R}}^3$ we have

$$\begin{aligned} {\mathcal {H}}^{0}(f^{-1}\{x_0\})\le \frac{1}{4\pi } {\mathcal {H}}_{c_0}(f) + \frac{c_0}{2\pi }\int \limits _{F[M]} \frac{{\mathcal {H}}^{0}(F^{-1}\{x\})}{|x-x_0|^{2}}\mathop {}\!\textrm{d}{\mathcal {L}}^3(x). \end{aligned}$$

In particular, in case $c_0\le 0$ we infer

$$\begin{aligned} {\mathcal {H}}^{0}(f^{-1}\{x_0\})\le \frac{1}{4\pi }{\mathcal {H}}_{c_0}(f). \end{aligned}$$

Due to round spheres, the above extension of (1.5) can only hold if $c_0\le 0$ and *n* is the inner unit normal. Of course, in view of (1.1) we could simply reverse the orientation on $\Sigma$, but this will generically make it impossible to find an Alexandrov immersion where *M* in Definition 1.4 is compact.

As a key application of our Li–Yau inequalities, we prove the following contribution to Problem 1.1 based on the previous result in [31].

#### Theorem 1.6

Let $c_0\in {\mathbb {R}}$ and suppose $A_0,V_0>0$ satisfy the isoperimetric inequality $36\pi V_0^2\le A_0^3$. Set

1.9 $$\begin{aligned} \eta (c_0, A_0, V_0) :=\inf \{ {\mathcal {H}}_{c_0}(f)\,\, f \in C^{\infty }({\mathbb {S}}^2;{\mathbb {R}}^3)\text { embedding, } {\mathcal {A}}(f)=A_0,{{\,\mathrm{{\mathcal {V}}}\,}}(f)=V_0\}. \end{aligned}$$

There exists $\Gamma (c_0, A_0, V_0) \in {\mathbb {R}}$ such that if

$$\begin{aligned} \eta (c_0, A_0, V_0) < 8\pi + \Gamma (c_0, A_0, V_0) \end{aligned}$$

then the infimum in (1.9) is attained. Moreover, there holds

$$\begin{aligned} \Gamma (c_0, A_0, V_0)\ge {\left\{ \begin{array}{ll} 4\pi \left( \sqrt{1 + \frac{\vert c_0\vert V_0}{2\cdot 9^2 (A_0 + \frac{2}{3}\vert c_0\vert V_0)}} - 1\right) &{} \text { if }c_0< 0, \\ -6c_0(4\pi ^2V_0)^\frac{1}{3}&{} \text { if }c_0\ge 0. \end{array}\right. } \end{aligned}$$

In particular $\Gamma (c_0, A_0, V_0)>0$ for $c_0<0$ and for any $c_0\le 0$ there exist $A_0, V_0>0$ with $\eta (c_0, A_0, V_0)<8\pi$.

The explicit estimates of the constant $\Gamma (c_0, A_0, V_0)$ are due to further geometric applications of our Li–Yau inequality, see Lemma 6.4 and Remark 6.11. As a consequence of Theorem 1.6, the only missing step to exclude bubbling in Problem 1.1 are estimates from above for $\eta (c_0, A_0, V_0)$. This is an interesting problem to be addressed in future research. One idea is to construct example surfaces that can be used to derive these bounds numerically.

### A suitable setup for the Li–Yau inequalities

We now discuss the different notions of (generalized) surfaces that we want to prove and apply Li–Yau inequalities for. There are four key points to be considered.

(i) In order to even define the Helfrich energy, the surface needs to have a unit normal vector field. In the smooth case, this naturally means that the surface is orientable.
(ii) One of the main applications of the classical Li–Yau inequality for the Willmore functional is to deduce embeddedness of immersions whose energy lies strictly below $8\pi$. Therefore, the Li–Yau inequality should hold for surfaces that are not already embedded a priori, i.e. we want to allow for multiplicity points.
(iii) In order to actually apply the Li–Yau inequality for the Helfrich energy (see Lemma 1.3), it is necessary to determine the sign of the concentrated volume (1.7). A sufficient tool to do so would be a divergence theorem.
(iv) Another important application of the classical Li–Yau inequality is to infer regularity and embeddedness of minimizers. It is therefore of interest to prove Li–Yau inequalities for weak surfaces that have good compactness properties.

**Fig. 2:**
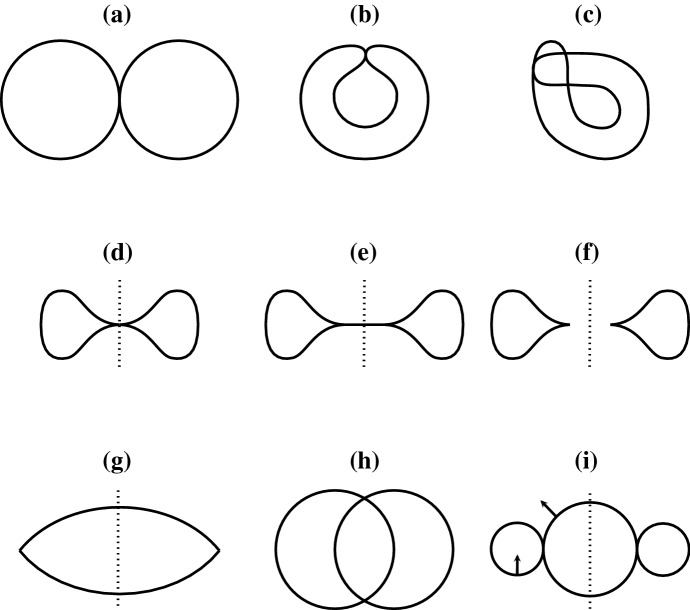
Profiles of surfaces with different types of multiplicity points. Dotted lines indicate rotationally symmetric surfaces

**Oriented varifolds** The most general notion of surface that comprises all shapes shown in Fig. 2a–i and that naturally satisfies Items (i), (ii), and (iv) are *oriented varifolds,* cf. [21]. They generalize the idea of immersed submanifolds and allow for a generalized concept of mean curvature. Since they also possess strong compactness properties, they have already been applied in several variational settings for the Canham–Helfrich model, see [8, 14, 15]. Our most general version of the Li–Yau inequality for oriented varifolds, Theorem 4.2, is also applicable if the first variation has a nontrivial singular part $\beta$ (see Hypothesis 2.1). The reason for this generality is that we would like the Li–Yau inequality to be applicable for surfaces like the one shown in Fig. 2g, see also Example 4.7. Moreover, the Li–Yau inequality can then also be applied in the context of boundary problems, see [14]. These are naturally formulated using *curvature varifolds with boundary,* cf. [26].

**Alexandrov immersions** In nature, one expects the principle of noninterpenetration of matter to hold true. As for vesicles that means there is a clearly defined inside. Nevertheless, membranes can be squeezed together as in Fig. 2b, d, e. In order to satisfy a divergence theorem, a surface should possess a well defined inside. In the smooth case, the so called *Alexandrov immersions* (see [1] and Definition 1.4 above) do satisfy a divergence theorem, see Lemma 5.1 below. They allow for multiplicity points as shown in Fig. 2b, c. Moreover, since the underlying 3-manifold of an Alexandrov immersion does not have to be connected, they also allow for multiplicity points that arise from two touching surfaces as shown in Fig. 2a or even two intersecting surfaces as shown in Fig. 2h. However, the rotationally symmetric surface in Fig. 2d is not an Alexandrov immersion. The Li–Yau inequality for Alexandrov immersions is stated in Theorem 1.5.

**Sets of finite perimeter** A nonsmooth notion of surfaces that satisfy a divergence theorem are the boundaries of *sets of finite perimeter,* cf. [17, Chapter 5]. As opposed to Alexandrov immersions, they allow for multiplicity points as shown in Fig. 2d but they do not allow for the multiplicity points in Fig. 2c. Sets of finite perimeter do have good compactness properties. Moreover, they comprise nonsmooth objects as shown in Fig. 2g and discussed in Example 4.7. In Sect. 4.3, we introduce a weak notion of Alexandrov immersions, the *varifolds with enclosed volume*. Their underlying 3-dimensional structure is a sequence of decreasing sets of finite perimeter. They allow for multiplicity points as in Fig. 2a–e and Fig. 2h and still satisfy a divergence theorem. The corresponding Li–Yau inequality is stated in Corollary 4.11.

**Currents** Another important class of surfaces that naturally satisfies Items (i) and (iv) above are *currents* (see [18, Chapter 4]). A downside of this concept is that the current associated with the immersion of Fig. 2e corresponds to the surface shown in Fig. 2f. More precisely, a current induced by an immersion with a given unit normal field looses information about multiplicity points that arise by overlapping where the sum of the unit normal vectors vanishes, cf. also (6.17). As a consequence, the varifold corresponding to the surface in Fig. 2f has a nontrivial singular part while the varifold corresponding to the immersion of Fig. 2e has no singular part.

**Lipschitz quasi-embeddings** In Sect. 6.3, we introduce a concept to model cellular membranes as weak immersions that can only self intersect tangentially. This is a new concept inspired by the previously developed *Lipschitz immersions* of Rivière [33]. The resulting class of surfaces is termed *Lipschitz quasi-embeddings* and satisfies Items (i)–(iv) above. They describe cellular shapes and comprise the surfaces in Fig. 2b, d, e, but do not allow for interpenetration as in Fig. 2c. It turns out that this class is well-suited for the variational discussion of the spherical Canham–Helfrich model which is why we rely on it for the proof of Theorem 1.6.

The only kind of surface where the sign of the concentrated volume cannot be determined in general are those surfaces where the unit normal vector field changes between inner and outer, see Fig. 2i and Example 3.8. These are surfaces where interpenetration necessarily happens.

### Structure of this article

After a brief discussion of the geometric and measure theoretic background in Sect. 2, we examine the concentrated volume and its properties in Sect. 3. This includes Hölder continuity in $x_0\in {\mathbb {R}}^3$ and continuity with respect to varifold convergence. In Sect. 4, we then derive a monotonicity formula for the Helfrich functional, from which we deduce our most general Li–Yau inequality for varifolds, Theorem 4.2. After that, we review the notion of sets of finite perimeter and introduce the concept of varifolds with enclosed volume. The Li–Yau inequalities in the smooth setting, Lemma 1.3 and Theorem 1.5 are then a direct application, see Sect. 5. Finally, in Sect. 6, we derive some geometric estimates and discuss implications of our results. This includes a nonexistence result for the penalized version of Problem 1.1 (Sect. 6.2), diameter bounds (Sect. 6.2), the existence and regularity result for the Canham–Helfrich model, Theorem 1.6 (Sect. 6.3), and a criterion for positive total mean curvature (Sect. 6.4).

## Preliminaries

In this section, we will review some of the concepts and tools used throughout this article.

### Notation and definitions

Let $\mu$ be a Radon measure over ${\mathbb {R}}^3$ and define the closed balls

$$\begin{aligned} B_\rho (x) :=\{y\in {\mathbb {R}}^3\mid |x-y|\le \rho \} \end{aligned}$$

for all $x\in {\mathbb {R}}^3$ and $\rho >0$. For each nonnegative integer *m* and $x\in {\mathbb {R}}^3$, the *m*-dimensional *lower* and *upper density* of $\mu$ at *x* are defined by

$$\begin{aligned} \theta ^{m}_*(\mu ,x) :=\liminf _{\rho \rightarrow 0+}\frac{\mu (B_\rho (x))}{\omega _m\rho ^m},\qquad \theta ^{*m}(\mu ,x) :=\limsup _{\rho \rightarrow 0+}\frac{\mu (B_\rho (x))}{\omega _m\rho ^m}, \end{aligned}$$

where $\omega _m = {\mathcal {L}}^m(B_1(0))$ and ${\mathcal {L}}^m$ is the *m*-dimensional Lebesgue measure over ${\mathbb {R}}^m$. The *m*-dimensional *density* of $\mu$ at *x* is defined by

$$\begin{aligned} \theta ^{m}(\mu ,x) :=\lim _{\rho \rightarrow 0+}\frac{\mu (B_\rho (x))}{\omega _m\rho ^m}, \end{aligned}$$

provided the limit exists. We define the *support* of $\mu$ by

$$\begin{aligned} {{\,\textrm{spt}\,}}\mu :={\mathbb {R}}^3 {\setminus }\{x \in {\mathbb {R}}^3\mid \exists \rho >0\text { such that }\mu (B_\rho (x)) = 0\}. \end{aligned}$$

The *m*-dimensional Hausdorff measure in Euclidean space is denoted with ${\mathcal {H}}^m$. We say that an integral exists if and only if it exists in the Lebesgue sense (i.e. its integrand is *summable* in the terminology of [18, 2.4.2]).

### Oriented 2-varifolds

Let ${\mathbb {G}}^{\mathrm o}(3,2)$ be the set of *oriented 2-dimensional subspaces of* ${\mathbb {R}}^3$. In view of [18, 3.2.28(2)], we identify ${\mathbb {G}}^{\mathrm o}(3,2)$ with

$$\begin{aligned} \{\xi \in \textstyle \bigwedge _2 {\mathbb {R}}^3 \mid \xi \text { is simple, }|\xi |=1\} \end{aligned}$$

which is a smooth submanifold of the 2nd exterior power $\bigwedge _2 {\mathbb {R}}^3$. In particular, ${\mathbb {G}}^{\mathrm o}(3,2)$ is a locally compact Hausdorff space.

Following Hutchinson [21], we say that *V* is an *oriented* 2-*varifold* on ${\mathbb {R}}^3$, if and only if *V* is a Radon measure over ${\mathbb {G}}^\textrm{o}_2({\mathbb {R}}^3):={\mathbb {R}}^3 \times {\mathbb {G}}^\textrm{o}(3,2)$. The *weight measure* $\mu _V$ on ${\mathbb {R}}^3$ is defined by

$$\begin{aligned} \mu _V(A) :=V\bigl (\{(x,\xi ) \in {\mathbb {G}}_2^\textrm{o}({\mathbb {R}}^3) \mid x \in A\}\bigr ) \qquad \text {whenever }A\subset {\mathbb {R}}^3. \end{aligned}$$

It is the push forward of *V* under the projection ${\mathbb {R}}^3 \times {\mathbb {G}}^\textrm{o}(3,2)\rightarrow {\mathbb {R}}^3$. The set of oriented 2-varifolds in ${\mathbb {R}}^3$ is denoted by $\mathbb V_2^\textrm{o}({\mathbb {R}}^3)$.

For each $\xi \in {\mathbb {G}}^{\mathrm o}(3,2)$ we define the unoriented 2-dimensional subspace $T(\xi )$ of ${\mathbb {R}}^3$ by

$$\begin{aligned} T(\xi ) :=\{v \in {\mathbb {R}}^3 \mid v \wedge \xi = 0\}. \end{aligned}$$

Since $\xi$ is simple, there exist $v_1,v_2\in {\mathbb {R}}^3$ such that $\xi = v_1\wedge v_2$. Moreover, $|\xi |=1$ implies that $v_1\wedge v_2 = e_1 \wedge e_2$ for $e_1:=v_1/|v_1|$ and $e_2:=\tilde{v}_2/|{{\tilde{v}}}_2|$ where ${{\tilde{v}}}_2:=v_2 - \langle e_1,v_2\rangle e_1$. In other words, each $\xi \in \mathbb G^\textrm{o}(3,2)$ corresponds to an oriented orthonormal basis $(e_1,e_2)$ with $\xi = e_1\wedge e_2$ and $T(\xi ) = {{\,\textrm{span}\,}}\{e_1,e_2\}$. In particular, each oriented 2-varifold $V\in {\mathbb {V}}_2^\textrm{o}({\mathbb {R}}^3)$ induces a general (unoriented) 2-varifold in the sense of [2, Definition 3.1], given by the push forward of *V* under the map $q(x,\xi ) :=(x,T(\xi ))$. Notice that the two weight measures of *V* and $q_\#V$ coincide.

For all compactly supported vector fields $X \in C^1_c({\mathbb {R}}^3;{\mathbb {R}}^3)$ and 2-dimensional subspaces *T* of ${\mathbb {R}}^3$ with orthonormal basis $\{e_1,e_2\}$, we define

$$\begin{aligned} {{\,\textrm{div}\,}}_T X(x) :=\sum _{j=1}^2 \langle e_j, \mathrm D X(x)e_j \rangle . \end{aligned}$$

The *first variation* of an oriented 2-varifold *V* in ${\mathbb {R}}^3$ is defined by

2.1 $$\begin{aligned} \delta V :C^1_c({\mathbb {R}}^3;{\mathbb {R}}^3) \rightarrow {\mathbb {R}}, \quad \delta V(X) :=\int \limits _{{\mathbb {G}}^\textrm{o}_2({\mathbb {R}}^3)} {{\,\textrm{div}\,}}_{T(\xi )} X(x) \mathop {}\!\textrm{d}V(x,\xi ). \end{aligned}$$

Notice that $\delta V$ coincides with the first variation of the unoriented 2-varifold $q_\#V$ as defined in [2, Definition 4.2]. In other words, $\delta V$ does not depend on the orientation.

For $k=0, \dots , 3$ we may identify *k*-vectors in ${\mathbb {R}}^3$ with $(3-k)$-vectors by means of the Hodge star operator

$$\begin{aligned} \star :\textstyle \bigwedge _k {\mathbb {R}}^3 \rightarrow \textstyle \bigwedge _{3-k} {\mathbb {R}}^3. \end{aligned}$$

If $v_1, v_2\in {\mathbb {R}}^3\simeq \textstyle \bigwedge _1{\mathbb {R}}^3$, we have $\star (v_1 \wedge v_2) = v_1 \times v_2$, where $\times$ denotes the usual cross product on ${\mathbb {R}}^3$. In particular, for all $\xi \in \mathbb G^\textrm{o}(3,2)$ there holds $|\star \xi | = 1$. Moreover, we have $\star \star v=v$ for all $v\in {\mathbb {R}}^3 \simeq \textstyle \bigwedge _1{\mathbb {R}}^3$.

If *V* has locally bounded first variation, that is the total variation of $\delta V$ is a Radon measure, then $\delta V$ can be represented by integration as follows, where the singular part will be denoted by $\beta _V$ (i.e. $\beta _V=\Vert \delta V \Vert _{\textrm{sing}}$, cf. [2, 4.3]).

#### Hypothesis 2.1

Let $V\in {\mathbb {V}}_2^\textrm{o}({\mathbb {R}}^3)$, $\eta \in L^{\infty }(\beta _V;{\mathbb {S}}^2)$, and $H \in L^1_\textrm{loc}(\mu _V;{\mathbb {R}}^3)$. Suppose

2.2 $$\begin{aligned} \delta V(X) = -\int \limits _{{\mathbb {R}}^3} \langle X, H\rangle \mathop {}\!\textrm{d}\mu _V + \int \limits _{{\mathbb {R}}^3} \langle X, \eta \rangle \mathop {}\!\textrm{d}\beta _V \end{aligned}$$

and

2.3 $$\begin{aligned} H(x)\wedge \star \xi = 0 \qquad \hbox {for} V \hbox {-almost all} (x,\xi ). \end{aligned}$$

The map *H* is often referred to as *generalized mean curvature* and ${{\,\textrm{spt}\,}}\beta _V$ can be seen as *generalized boundary*. Indeed, one can understand $\beta _V$ as the boundary measure. However, two boundary parts can fall together as in Fig. 2g. Typically, one can determine *H* and $\beta _V$ using Remark 4.4 and 4.7 in [2]. If *V* is rectifiable (i.e. $q_\#V$ is rectifiable in the sense of [2, 3.5]) then the condition in (2.3) means that the generalized mean curvature is perpendicular. It is satisfied provided *V* is an integral varifold, see [7, Section 5.8]. In the absence of the singular part, Hypothesis 2.1 simplifies as follows.

#### Hypothesis 2.2

Let $V\in {\mathbb {V}}_2^\textrm{o}({\mathbb {R}}^3)$ and $H \in L^1_\textrm{loc}(\mu _V;{\mathbb {R}}^3)$. Suppose

2.4 $$\begin{aligned} \delta V(X) = -\int \limits _{{\mathbb {R}}^3} \langle X, H\rangle \mathop {}\!\textrm{d}\mu _V \end{aligned}$$

and

$$\begin{aligned} H(x)\wedge \star \xi = 0 \qquad \text {for }V\text {-almost all }(x,\xi ). \end{aligned}$$

Let $c_0$ be a real number, and assume $V\in {{\mathbb {V}}}_2^\textrm{o}({\mathbb {R}}^3)$ and $H \in L^1_\textrm{loc}(\mu _V;{\mathbb {R}}^3)$ satisfy Hypothesis 2.1 for some $\eta \in L^\infty (\beta _V;{\mathbb {S}}^2)$. Then we define the *Helfrich energy*

$$\begin{aligned} {\mathcal {H}}_{c_0}(V) :=\frac{1}{4}\int |H(x) - c_0(\star \xi )|^2\mathop {}\!\textrm{d}V(x,\xi ) = \frac{1}{4}\int (\langle H(x), \star \xi \rangle - c_0)^2 \mathop {}\!\textrm{d}V(x,\xi ). \end{aligned}$$

Notice that the Helfrich energy does not depend on the singular part of the first variation. This is analogous to the definition in [14, Section 2]. For $c_0 = 0$, we obtain the *Willmore functional* ${\mathcal {W}}:={\mathcal {H}}_{0}$.

#### Remark 2.3

Since $\mu _V$ and *V* are Radon measures, we have $H\in L^2_\textrm{loc}(\mu _V;{\mathbb {R}}^3)$ if and only if the function $(x,\xi )\mapsto H(x) - c_0(\star \xi )$ is a member of $L^2_\textrm{loc}(V;{\mathbb {R}}^3)$. Indeed, given any Borel set *B* in ${\mathbb {R}}^3$, the Cauchy–Schwarz inequality implies

$$\begin{aligned} \int \limits _{B\times {\mathbb {G}}^\textrm{o}(3,2)} |H(x) - c_0(\star \xi )|^2\mathop {}\!\textrm{d}V(x,\xi ) \le 2\int \limits _B |H|^2\mathop {}\!\textrm{d}\mu _V +2 c_0^2 \mu _V(B). \end{aligned}$$

On the other hand,

2.5 $$\begin{aligned} \int \limits _B |H|^2\mathop {}\!\textrm{d}\mu _V&\le \int \limits _{B\times \mathbb G^\textrm{o}(3,2)} 2|H(x) - c_0(\star \xi )|^2\mathop {}\!\textrm{d}V(x,\xi ) + 2c_0^2\mu _V(B). \end{aligned}$$

In particular, ${\mathcal {H}}_{c_0}(V)<\infty$ implies $H\in L^2_\textrm{loc}(\mu _V;{\mathbb {R}}^3)$.

### Oriented varifolds induced by immersions

A particular class of oriented varifolds will be given by immersions of oriented surfaces. Following [36], we term a surface $\Sigma$ to be *orientable*, if there exists an atlas $A = \{(U_\alpha ,x_\alpha )\}_{\alpha \in I}$ such that the Jacobians $\det \mathrm D(x_{\alpha _1}\circ x_{\alpha _2}^{-1})$ of all coordinate transformations are positive. The members of *A* are called *positive* charts. If $f:\Sigma \rightarrow {\mathbb {R}}^3$ is a smooth immersion, then we define the induced smooth normal field *n* along *f* (the *Gauss map*) by

2.6 $$\begin{aligned} n:\Sigma \rightarrow {\mathbb {S}}^2, \quad n :=\frac{\partial _{x^1}f \times \partial _{x^2}f}{|\partial _{x^1}f \times \partial _{x^2}f|}, \end{aligned}$$

whenever *x* is a positive chart. Notice that since the Hodge star operator is an isometry, $\star n = \partial _{x^1}f\wedge \partial _{x^2}f/|\partial _{x^1}f\wedge \partial _{x^2}f|$ takes values in ${\mathbb {G}}^\textrm{o}(3,2)$. Moreover, in the context of an immersion *f*, we will always denote by $\mu =\mu _f$ the Riemannian measure induced by the pullback metric $g=g_f:=f^*\langle \cdot ,\cdot \rangle$, and we define by

$$\begin{aligned} {\mathcal {A}}(f) :=\int \limits _{\Sigma }1\mathop {}\!\textrm{d}\mu ,\quad {{\,\mathrm{{\mathcal {V}}}\,}}(f) :=-\frac{1}{3}\int \limits _{\Sigma }\langle f, n\rangle \mathop {}\!\textrm{d}\mu \end{aligned}$$

the area and the (algebraic) volume of *f*, provided the respective integral exists. If *f* is an embedding and *n* is the inner unit normal, ${{\,\mathrm{{\mathcal {V}}}\,}}(f)$ yields the enclosed volume as a consequence of the divergence theorem, see [34, Appendix A] for a more detailed discussion.

In the sequel, the immersion under consideration will usually be clear from the context, so we will drop the dependence on *f* of the associated geometric quantities.

#### Example 2.4

(Oriented varifold associated with immersed surface) Let $f:\Sigma \rightarrow {\mathbb {R}}^3$ be a smooth proper immersion of an oriented surface $\Sigma$ without boundary. We define the oriented 2-varifold $V \in {\mathbb {V}}_2^\textrm{o}({\mathbb {R}}^3)$ associated with $(\Sigma ,f)$ by

$$\begin{aligned} V(A) :=\mu \bigl (\{p \in \Sigma \mid (f(p), \star n(p)) \in A \}\bigr ) \qquad \text {whenever }A\subset \mathbb {G}^\textrm{o}_2({\mathbb {R}}^3), \end{aligned}$$

i.e. *V* is the push forward of $\mu$ under the map $\Sigma \rightarrow {\mathbb {R}}^3 \times {\mathbb {G}}^\textrm{o}(3,2), p\mapsto (f(p), \star n(p))$. Since this map is continuous and proper, *V* is indeed a Radon measure (see [18, 2.2.17]). Notice that $T(\star n(p)) = \mathrm d f_p[T_p\Sigma ]$ for $p\in \Sigma$. In view of [39, Lemma 2.3], there holds

$$\begin{aligned} \mu _V(B) = (f_\#\mu )(B) = \int \limits _B{\mathcal {H}}^0(f^{-1}\{x\})\mathop {}\!\textrm{d}{\mathcal {H}}^2(x) \qquad \text {for all Borel sets }B\text { in }{\mathbb {R}}^3,\\ \theta ^2(\mu _V,x) = {\mathcal {H}}^0(f^{-1}\{x\}) \qquad \text {for all }x\in {\mathbb {R}}^3. \end{aligned}$$

Moreover, by [18, 2.4.18] and the area formula (cf. [39, Lemma 2.3]), we have

2.7 $$\begin{aligned} \int \limits _{{\mathbb {G}}_2^\textrm{o}({\mathbb {R}}^3)} k(x,\xi )\mathop {}\!\textrm{d}V(x,\xi )&= \int \limits _\Sigma k(f(p),\star n(p))\mathop {}\!\textrm{d}\mu (p) \nonumber \\&= \int \limits _{{\mathbb {R}}^3}\sum _{p\in f^{-1}\{x\}} k(x, \star n(p))\mathop {}\!\textrm{d}{\mathcal {H}}^2(x) \end{aligned}$$

whenever $k:{\mathbb {G}}_2^\textrm{o}({\mathbb {R}}^3)\rightarrow {\mathbb {R}}$ is a nonnegative Borel function.

Let $H_f:\Sigma \rightarrow {\mathbb {R}}^3$ be the classical mean curvature (vector) of *f*, i.e. the trace of the second fundamental form, and define

2.8 $$\begin{aligned} H(x) :={\left\{ \begin{array}{ll} \frac{1}{\theta ^2(\mu _V,x)}\sum _{p\in f^{-1}\{x\}}H_f(p) &{} \text {if }\theta ^2(\mu _V,x) >0 \\ 0&{}\text {if }\theta ^2(\mu _V,x) =0. \end{array}\right. } \end{aligned}$$

Then, $H\in L^\infty _{\textrm{loc}}(\mu _V;{\mathbb {R}}^3)$ and in view of [39, Example 2.4], $H(x)\wedge \star \xi = 0$ for *V*-almost all $(x,\xi )$, and

$$\begin{aligned} \delta V(X) = -\int \limits _{{\mathbb {R}}^3} \langle X,H\rangle \mathop {}\!\textrm{d}\mu _V. \end{aligned}$$

Thus, *V*, *H* satisfy Hypothesis 2.2.

In the sequel, we will always use the above notation to distinguish $H_f$ as the classical mean curvature when *f* is an immersion and *H* defined by (2.8) as the generalized mean curvature of the associated varifold. By [40, Theorem 4], there holds

$$\begin{aligned} H_f(p) = H(f(p))\quad \text {for }\mu \text {-almost all }p\in \Sigma . \end{aligned}$$

Thus, by (2.7) we observe

2.9 $$\begin{aligned} {\mathcal {H}}_{c_0}(V) = \frac{1}{4}\int \limits _{\Sigma } |H_f - c_0n|^2\mathop {}\!\textrm{d}\mu . \end{aligned}$$

## On the concentrated volume

In this section, we discuss the concentrated volume (1.7) in the context of varifolds.

### Definition 3.1

Suppose $V\in {\mathbb {V}}_2^\textrm{o}({\mathbb {R}}^3)$ and $x_0\in {\mathbb {R}}^3$. Then we define the *concentrated volume of* *V* *at* $x_0$ by

$$\begin{aligned} {{\,\mathrm{{\mathcal {V}}}\,}}_c(V,x_0) :=- \int \limits _{\mathbb G_2^\textrm{o}({\mathbb {R}}^3)}\frac{\langle x-x_0,\star \xi \rangle }{|x-x_0|^2}\mathop {}\!\textrm{d}V(x,\xi ) \end{aligned}$$

and the *algebraic volume at* $x_0$

$$\begin{aligned} {{\,\mathrm{{\mathcal {V}}}\,}}(V,x_0) :=- \frac{1}{3}\int \limits _{\mathbb G_2^\textrm{o}({\mathbb {R}}^3)}\langle x-x_0,\star \xi \rangle \mathop {}\!\textrm{d}V(x,\xi ) \end{aligned}$$

provided the respective integral exists.

If the varifold *V* is associated with an immersion $f:\Sigma \rightarrow {\mathbb {R}}^3$, then we also write ${{\,\mathrm{{\mathcal {V}}}\,}}_c(f,x_0)$ instead of ${{\,\mathrm{{\mathcal {V}}}\,}}_c(V,x_0)$. By (2.7), this is consistent with (1.7). If $\Sigma$ is closed, then we have ${{\,\mathrm{{\mathcal {V}}}\,}}(V,x_0)={{\,\mathrm{{\mathcal {V}}}\,}}(f)$ for all $x_0\in {\mathbb {R}}^3$ after integration by parts.

In general, the algebraic volume of an oriented varifold depends on the point $x_0$. Indeed, one may consider the varifold associated to the 2-dimensional unit sphere in ${\mathbb {R}}^3$ where the upper hemisphere is oppositely oriented to the lower hemisphere. Moreover, the algebraic volume at $x_0$ exists if and only if the concentrated volume at $x_0$ exists, see Proposition 3.4.

### Lemma 3.2

Suppose $m,\rho _0,D>0$, $\mu$ is a Radon measure over ${\mathbb {R}}^3$, $x_0\in {\mathbb {R}}^3$, and

$$\begin{aligned} \mu (B_\sigma (x_0)) \le D\sigma ^m \end{aligned}$$

for all $0<\sigma <\rho _0$. Then, for all $1\le p <m$, there exists $C(p,m,D) <\infty$ such that

$$\begin{aligned} \int \limits _{B_\sigma (x_0)}\frac{1}{|x-x_0|^p}\mathop {}\!\textrm{d}\mu (x) \le C(p,m,D) \sigma ^{m-p} \end{aligned}$$

for all $0<\sigma <\rho _0$. Moreover, $C(1,2,D) = 2D$, and if $\mu ({\mathbb {R}}^3)<\infty$ then

$$\begin{aligned} \int \limits _{{\mathbb {R}}^3}\frac{1}{|x-x_0|^p}\mathop {}\!\textrm{d}\mu (x) \le C(p,m,D) \rho _0^{m-p} + \frac{\mu ({\mathbb {R}}^3)}{\rho _0^p} < \infty . \end{aligned}$$

### Remark 3.3

(i) Assume $V\in {\mathbb {V}}_2^\textrm{o}({\mathbb {R}}^3)$, $H\in L^{2}_{\textrm{loc}}(\mu _V;{\mathbb {R}}^3)$ satisfy Hypothesis 2.2. By [39, Theorem 3.6], we find that the density $\theta ^2(\mu _V,x_0)$ exists and is finite for all $x_0\in {\mathbb {R}}^3$. Hence there exist $\rho _0=\rho _0(V,x_0)>0$ and $D=D(V,x_0)<\infty$ such that
  $$\begin{aligned} \mu _V(B_\sigma (x_0))\le D\sigma ^2 \quad \text { for all }0<\sigma <\rho _0, x_0\in {\mathbb {R}}^3. \end{aligned}$$
  This immediately implies $\mu _V(\{x_0\})=0$ for all $x_0\in {\mathbb {R}}^3$. By Remark 2.3, the condition $H\in L^2_{\textrm{loc}}(\mu _V;{\mathbb {R}}^3)$ is in particular satisfied if ${\mathcal {H}}_{c_0}(V)<\infty$.
(ii) If $V\in {\mathbb {V}}_2^\textrm{o}({\mathbb {R}}^3)$, $\mu _V({\mathbb {R}}^3)<\infty$, and $H\in L^2(\mu _V;{\mathbb {R}}^3)$ satisfies Hypothesis 2.2, then the hypothesis of Lemma 3.2 is satisfied for $m=2$, $\rho _0 =\infty$, and all $x_0 \in {\mathbb {R}}^3$ with $D = C{\mathcal {W}}(V)$ for some universal constant $0<C<\infty$. Indeed, by [24, Appendix (A.16)] there holds
  $$\begin{aligned} \mu _V(B_\sigma (x_0)) \le C{\mathcal {W}}(V) \sigma ^2 \quad \text { for all }\sigma >0, x_0\in {\mathbb {R}}^3. \end{aligned}$$

### Proof of Lemma 3.2

Using Fubini’s theorem, we compute (cf. [27, Theorem 1.15])

$$\begin{aligned} \int \limits _{B_\sigma (x_0)}\frac{1}{|x-x_0|^p}\mathop {}\!\textrm{d}\mu (x)&= \int \limits _0^\infty \mu (B_\sigma (x_0)\cap B_{t^{-1/p}}(x_0))\mathop {}\!\textrm{d}t \\&=\int \limits _0^{\sigma ^{-p}}\mu (B_\sigma (x_0))\mathop {}\!\textrm{d}t + \int \limits _{\sigma ^{-p}}^\infty \mu (B_{t^{-1/p}}(x_0))\mathop {}\!\textrm{d}t \\&\le D\left( \int \limits _0^{\sigma ^{-p}}\sigma ^m\mathop {}\!\textrm{d}t + \int \limits _{\sigma ^{-p}}^\infty t^{-m/p}\mathop {}\!\textrm{d}t\right) \\&=D\left( \sigma ^{m-p} + \frac{p\sigma ^{m-p}}{m-p}\right) = C(p,m,D)\sigma ^{m-p}. \end{aligned}$$

The last statement follows by splitting the integral into ${\mathbb {R}}^3 = B_{\rho _0}(x_0) \cup ({\mathbb {R}}^3 {\setminus } B_{\rho _0}(x_0))$. $\square$

### Proposition 3.4

Suppose $V\in {\mathbb {V}}^{\textrm{o}}_2({\mathbb {R}}^3)$ and $H\in L^2_{\textrm{loc}}(\mu _V;{\mathbb {R}}^3)$ satisfy Hypothesis 2.2 and assume that ${{\,\mathrm{{\mathcal {V}}}\,}}(V,x_0)$ exists for some $x_0\in {\mathbb {R}}^3$. Then also ${{\,\mathrm{{\mathcal {V}}}\,}}_c(V,x_0)$ exists.

### Proof

Splitting the integral, for $\rho _0>0$ we find

$$\begin{aligned}&\int \limits _{{\mathbb {G}}^\textrm{o}_2({\mathbb {R}}^3)}\frac{|\langle x-x_0, \star \xi \rangle |}{|x-x_0|^2}\mathop {}\!\textrm{d}V(x,\xi ) \le \int \limits _{B_{\rho _0}(x_0)}\frac{1}{|x-x_0|}\mathop {}\!\textrm{d}\mu _V(x) + \frac{1}{\rho _0^2}\int \limits _{{\mathbb {G}}^{\textrm{o}}_2({\mathbb {R}}^3)}|\langle x-x_0, \star \xi \rangle |\mathop {}\!\textrm{d}V(x,\xi ). \end{aligned}$$

By Lemma 3.2 and Remark 3.3(i), on the right hand side the first integral is finite for $\rho _0>0$ small, whereas the second integral is finite since ${{\,\mathrm{{\mathcal {V}}}\,}}(V,x_0)$ exists. $\square$

We recall the concept of convergence of oriented varifolds.

### Definition 3.5

Suppose $V_k$ is a sequence in ${\mathbb {V}}_2^\textrm{o}({\mathbb {R}}^3)$. Then we say that $V_k$ *converges to* *V* *in* $\mathbb V_2^\textrm{o}({\mathbb {R}}^3)$ and write

$$\begin{aligned} V_k\rightarrow V\qquad \text {in }{\mathbb {V}}_2^\textrm{o}({\mathbb {R}}^3)\text { as }k\rightarrow \infty \end{aligned}$$

if and only if $V\in {\mathbb {V}}_2^\textrm{o}({\mathbb {R}}^3)$ and

$$\begin{aligned} \int \limits _{{\mathbb {G}}_2^\textrm{o}({\mathbb {R}}^3)}\varphi (x,\xi )\mathop {}\!\textrm{d}V_k(x,\xi )\rightarrow \int \limits _{{\mathbb {G}}_2^\textrm{o}({\mathbb {R}}^3)}\varphi (x,\xi )\mathop {}\!\textrm{d}V(x,\xi ) \qquad \text {as }k\rightarrow \infty \end{aligned}$$

for all continuous functions $\varphi :\mathbb G_2^\textrm{o}({\mathbb {R}}^3)\rightarrow {\mathbb {R}}$ with compact support.

### Lemma 3.6

Suppose $V_k$ is a sequence in ${\mathbb {V}}_2^\textrm{o}({\mathbb {R}}^3)$, $V \in {\mathbb {V}}_2^\textrm{o}({\mathbb {R}}^3)$, $H_k\in L^2(\mu _{V_k};{\mathbb {R}}^3)$ and $H\in L^2(\mu _V;{\mathbb {R}}^3)$ satisfy Hypothesis 2.2,

3.1 $$\begin{aligned} \sup _{k\in {\mathbb {N}}} \left( \mu _{V_k}({\mathbb {R}}^3)+{\mathcal {W}}(V_k)\right) <\infty \end{aligned}$$

and

3.2 $$\begin{aligned} V_k\rightarrow V\qquad \text {in }{\mathbb {V}}_2^\textrm{o}({\mathbb {R}}^3)\text { as }k\rightarrow \infty . \end{aligned}$$

Then for all $x_0\in {\mathbb {R}}^3$, the concentrated volume converges: $\lim _{k\rightarrow \infty }{{\,\mathrm{{\mathcal {V}}}\,}}_c(V_k,x_0)={{\,\mathrm{{\mathcal {V}}}\,}}_c(V,x_0)$.

### Proof

Let $x_0\in {\mathbb {R}}^3$, $0<\sigma<\rho <\infty$, and pick a continuous function $\chi :{\mathbb {R}}^3\rightarrow {\mathbb {R}}$ with compact support in ${\mathbb {R}}^3{\setminus }\{x_0\}$ such that $0\le \chi \le 1$ and $\chi (x) = 1$ for $\sigma \le |x - x_0|\le \rho$. Define the function

$$\begin{aligned} \varphi :{\mathbb {R}}^3\times {\mathbb {G}}^\textrm{o}(3,2)\rightarrow {\mathbb {R}}, \qquad \varphi (x,\xi ) :=\chi (x) \frac{\langle x-x_0,\star \xi \rangle }{|x-x_0|^2}. \end{aligned}$$

Then $\varphi$ has compact support, $\varphi$ is continuous, and thus, by (3.2),

3.3 $$\begin{aligned} \int \limits _{{\mathbb {G}}_2^\textrm{o}({\mathbb {R}}^3)}\varphi \mathop {}\!\textrm{d}V_k \rightarrow \int \limits _{{\mathbb {G}}_2^\textrm{o}({\mathbb {R}}^3)}\varphi \mathop {}\!\textrm{d}V \qquad \text {as }k\rightarrow \infty . \end{aligned}$$

Let

$$\begin{aligned} A:=\sup _{k\in {\mathbb {N}}}\bigl (\mu _{V_k}({\mathbb {R}}^3)+\mu _V({\mathbb {R}}^3)\bigr ),\qquad D:=\sup _{k\in {\mathbb {N}}}\bigl ({\mathcal {W}}(V_k) + {\mathcal {W}}(V)\bigr ). \end{aligned}$$

Then, by (3.1), Lemma 3.2, and Remark 3.3(ii), we have

$$\begin{aligned} \sup _{k\in {\mathbb {N}}}\left( \int \limits _{B_\sigma (x_0)}\frac{1}{|x-x_0|}\mathop {}\!\textrm{d}\mu _{V_k}(x)+ \int \limits _{B_\sigma (x_0)}\frac{1}{|x-x_0|}\mathop {}\!\textrm{d}\mu _V(x)\right) \le C(D)\sigma \end{aligned}$$

and

$$\begin{aligned} \sup _{k\in {\mathbb {N}}}\left( \int \limits _{{\mathbb {R}}^3{\setminus } B_\rho (x_0)}\frac{1}{|x-x_0|}\mathop {}\!\textrm{d}\mu _{V_k}(x)+\int \limits _{{\mathbb {R}}^3{\setminus } B_\rho (x_0)}\frac{1}{|x-x_0|}\mathop {}\!\textrm{d}\mu _V(x)\right) \le \frac{C(A)}{\rho }. \end{aligned}$$

Since $|\varphi (x,\xi )|\le 1/|x-x_0|$ for all $(x,\xi )\in {\mathbb {G}}_2^\textrm{o}({\mathbb {R}}^3)$, it follows

$$\begin{aligned} |{{\,\mathrm{{\mathcal {V}}}\,}}_c(V,x_0) - {{\,\mathrm{{\mathcal {V}}}\,}}_c(V_k,x_0)|\le \left| \int \limits _{\mathbb G_2^\textrm{o}({\mathbb {R}}^3)}\varphi \mathop {}\!\textrm{d}V_k - \int \limits _{\mathbb G_2^\textrm{o}({\mathbb {R}}^3)}\varphi \mathop {}\!\textrm{d}V\right| +C(D)\sigma + \frac{C(A)}{\rho }. \end{aligned}$$

Now, the conclusion follows from the convergence in (3.3). $\square$

### Lemma 3.7

Suppose $V\in {\mathbb {V}}_2^\textrm{o}({\mathbb {R}}^3)$, ${{\,\textrm{spt}\,}}\mu _V$ is compact, and $H\in L^2(\mu _V;{\mathbb {R}}^3)$ satisfies Hypothesis 2.2. Then the concentrated volume ${{\,\mathrm{{\mathcal {V}}}\,}}_c(V,\cdot )$ is Hölder continuous with exponent $\alpha$ for any $0<\alpha <1$ and constant $C=C(\alpha ,V)$ depending monotonically nondecreasing on $\mu _V({\mathbb {R}}^3)$ and ${\mathcal {W}}(V)$.

### Proof

Let $0<\alpha <1$, $x_0,x_1\in {\mathbb {R}}^3$ with $0<|x_0-x_1|\le 1$, and abbreviate $\sigma :=|x_0-x_1|$, $A:=\mu _V({\mathbb {R}}^3)$ and $D:={\mathcal {W}}(V)$. By Lemma 3.2 and Remark 3.3(ii) there holds

$$\begin{aligned} \int \limits _{B_{2\sigma }(x_0)}\frac{1}{|x-x_0|}\mathop {}\!\textrm{d}\mu _V(x) \le 4D|x_0-x_1|\le C(D)|x_0-x_1|^\alpha \end{aligned}$$

and, since $$B_{2\sigma }(x_0)\subset B_{3\sigma }(x_1)$$,

$$\begin{aligned} \int \limits _{B_{2\sigma }(x_0)}\frac{1}{|x-x_1|}\mathop {}\!\textrm{d}\mu _V(x) \le \int \limits _{B_{3\sigma }(x_1)}\frac{1}{|x-x_1|}\mathop {}\!\textrm{d}\mu _V(x) \le 6D|x_0-x_1|\le C(D)|x_0-x_1|^\alpha . \end{aligned}$$

Thus, we have

3.4 $$\begin{aligned}&|{{\,\mathrm{{\mathcal {V}}}\,}}_c(V,x_0) - {{\,\mathrm{{\mathcal {V}}}\,}}_c(V,x_1)|\nonumber \\&\quad \le C(D)|x_0-x_1|^\alpha + \int \limits _{\pi ^{-1}[{\mathbb {R}}^3{\setminus } B_{2\sigma }(x_0)]}\left| \frac{\langle x-x_0,\star \xi \rangle }{|x-x_0|^2} - \frac{\langle x-x_1,\star \xi \rangle }{|x-x_1|^2}\right| \mathop {}\!\textrm{d}V(x,\xi ). \end{aligned}$$

where $\pi :{\mathbb {R}}^3\times {\mathbb {G}}^\textrm{o}(3,2)\rightarrow {\mathbb {R}}^3$ is the projection. For all $x\in {\mathbb {R}}^3{\setminus } B_{2\sigma }(x_0)$ we have

$$\begin{aligned} 2|x_0-x_1| = 2\sigma \le |x-x_0|, \qquad |x-x_0|\le 2|x-x_0| - 2|x_0-x_1| \le 2|x-x_1| \end{aligned}$$

and thus $|x-x_1|\ge |x-x_0|/2$. Since also $|x_0-x_1|\le |x-x_0|$, we infer

$$\begin{aligned}&\left| \frac{\langle x-x_0,\star \xi \rangle }{|x-x_0|^2} - \frac{\langle x-x_1,\star \xi \rangle }{|x-x_1|^2}\right| \\&\quad \le \left| \frac{\langle x-x_0,\star \xi \rangle }{|x-x_0|^2} - \frac{\langle x-x_1,\star \xi \rangle }{|x-x_0|^2}\right| + \left| \frac{\langle x-x_1,\star \xi \rangle }{|x-x_0|^2} - \frac{\langle x-x_1,\star \xi \rangle }{|x-x_0||x-x_1|}\right| \\&\quad \quad + \left| \frac{\langle x-x_1,\star \xi \rangle }{|x-x_0||x-x_1|} - \frac{\langle x-x_1,\star \xi \rangle }{|x-x_1|^2}\right| \\&\quad \le 2\frac{|x_0 - x_1|}{|x-x_0|^2} + \frac{|x_0 - x_1|}{|x-x_0||x-x_1|} \le 4\frac{|x_0-x_1|^\alpha }{|x-x_0|^{1+\alpha }}. \end{aligned}$$

Integrating this inequality and applying Lemma 3.2 for $p=1+\alpha$, $m=2$ and $\rho _0 = 1$, (3.4) becomes

$$\begin{aligned} |{{\,\mathrm{{\mathcal {V}}}\,}}_c(V,x_0) - {{\,\mathrm{{\mathcal {V}}}\,}}_c(V,x_1)|&\le C(D)|x_0-x_1|^\alpha + 4|x_0-x_1|^\alpha \int \limits _{{\mathbb {R}}^3}\frac{1}{|x-x_0|^{1+\alpha }}\mathop {}\!\textrm{d}\mu _V(x) \\&\le \bigl [C(D) + 4C(\alpha ,D) + 4A\bigr ]|x_0-x_1|^\alpha = C(\alpha ,A,D)|x_0-x_1|^\alpha . \end{aligned}$$

For $|x_0-x_1|\ge 1$ we apply Lemma 3.2 to see

$$\begin{aligned} |{{\,\mathrm{{\mathcal {V}}}\,}}_c(V,x_0) - {{\,\mathrm{{\mathcal {V}}}\,}}_c(V,x_1)| \le 2(2D + A) \le C(A,D) |x_0-x_1|^\alpha \end{aligned}$$

which concludes the proof. $\square$

### Example 3.8

Consider $S:=\partial B_1(0)\subset {\mathbb {R}}^3$, the round sphere with radius one centered at the origin. Moreover, for $r>0$ let $T_r\subset {\mathbb {R}}^3$ be the torus which is obtained by revolving a circle with radius *r* and center $(1+r,0)$ (in the *xz*-plane) around the *z*-axis. Note that if we revolve the corresponding disk instead of the circle, we obtain a full torus $T_r^{\textrm{full}}$ with $\partial T_r^{\textrm{full}} = T_r$. We now define a smooth unit normal *n* on $S\cup T_r$ by taking *n* to be the outer unit normal on *S* and the inner unit normal on $T_r$, cf. Fig. 2i. It is not difficult to see that $S\cup T_r$ is the image of a $C^{1,1}$-immersion $f:{\mathbb {S}}^2\rightarrow {\mathbb {R}}^3$.

By standard formulas in geometry, the algebraic volume can be computed as

3.5 $$\begin{aligned} {{\,\mathrm{{\mathcal {V}}}\,}}(f) = -{\mathcal {L}}^{3}(B_1(0)) + {\mathcal {L}}^{3}(T_r^{\textrm{full}}) = -\frac{4\pi }{3}+2\pi ^2 r^2(1+r). \end{aligned}$$

Let $x_0 = 0$ be the origin. Using that $|x-x_0|=1$ for $x\in S$ and applying the divergence theorem to $T_r^{\textrm{full}}$, the concentrated volume is given by

$$\begin{aligned} {{\,\mathrm{{\mathcal {V}}}\,}}_c(f,x_0) =-\frac{4\pi }{3} + \int \limits _{T_r^{\textrm{full}}} \frac{1}{|x-x_0|^2}\mathop {}\!\textrm{d}{\mathcal {L}}^3(x). \end{aligned}$$

Clearly $|x-x_0|>1$ for ${\mathcal {L}}^3$-almost every $x\in T_r^{\textrm{full}}$, so that

$$\begin{aligned} {{\,\mathrm{{\mathcal {V}}}\,}}_c(f,x_0)< -\frac{4\pi }{3} + \int \limits _{T_r^{\textrm{full}}}\mathop {}\!\textrm{d}{\mathcal {L}}^3(x) = {{\,\mathrm{{\mathcal {V}}}\,}}(f). \end{aligned}$$

By means of (3.5), we thus find $r>0$ such that ${{\,\mathrm{{\mathcal {V}}}\,}}(f)=0$ but ${{\,\mathrm{{\mathcal {V}}}\,}}_c(f,x_0)<0$. Slightly increasing the radius, we have ${{\,\mathrm{{\mathcal {V}}}\,}}_c(f,x_0)<0<{{\,\mathrm{{\mathcal {V}}}\,}}(f)$ by a continuity argument. Lastly, we replace a small disk on *S* by a thin dent, such that the new surface ${\tilde{S}}$ satisfies $x_0\in {\tilde{S}}$ and such that ${\tilde{S}}\cup T_r$ is still the image of an immersion ${\tilde{f}}:{\mathbb {S}}^2\rightarrow {\mathbb {R}}^3$. Making this dent sufficiently thin and smoothing, we can achieve that ${{\,\mathrm{{\mathcal {V}}}\,}}_c({\tilde{f}},x_0)<0<{{\,\mathrm{{\mathcal {V}}}\,}}({\tilde{f}})$ is still satisfied and ${\tilde{f}}$ is smooth. Therefore, positive algebraic volume does not imply positive concentrated volume, even at points in the support.

## The Li–Yau inequality in the varifold setting

### A monotonicity formula

Our first essential observation is the following lemma, which can be seen as an extension of the monotonicity formula due to Simon [43, (1.2)]. We follow the varifold approach in [24, Appendix A] relying on the first variation identity and examine the additional terms originating from the spontaneous curvature.

#### Lemma 4.1

Suppose $V \in {\mathbb {V}}_2^\textrm{o}({\mathbb {R}}^3)$, $\eta \in L^\infty (\beta _V;{\mathbb {S}}^2)$, and $H \in L^2_{\textrm{loc}}(\mu _V;{\mathbb {R}}^3)$ satisfy Hypothesis 2.1. Let $x_0\in {\mathbb {R}}^3$ and abbreviate $B_r:=B_r(x_0)$, $A_r:=B_r\times {\mathbb {G}}^\textrm{o}(3,2)$ for all $r>0$. Then, for $c_0\in {\mathbb {R}}$ and $0<\sigma<\rho <\infty$, there holds

4.1 $$\begin{aligned}&\frac{\mu _V(B_\sigma )}{\sigma ^2} + \int \limits _{A_{\rho }{\setminus } A_{\sigma }} \left( \frac{1}{4}(\langle H(x),\star \xi \rangle - c_0) + \frac{\langle x-x_0, \star \xi \rangle }{|x-x_0|^2}\right) ^2\mathop {}\!\textrm{d}V(x,\xi )\nonumber \\&\quad = \frac{1}{16}\int \limits _{A_{\rho }{\setminus } A_{\sigma }}|H(x) - c_0(\star \xi )|^2\mathop {}\!\textrm{d}V(x,\xi ) - \frac{c_0}{2}\int \limits _{A_{\rho }{\setminus } A_{\sigma }}\frac{\langle x-x_0,\star \xi \rangle }{|x-x_0|^2}\mathop {}\!\textrm{d}V(x,\xi ) + \frac{\mu _V(B_\rho )}{\rho ^2}\nonumber \\&\quad \quad - \frac{1}{2\sigma ^2}\int \limits _{A_\sigma }\langle x-x_0,H(x)-c_0(\star \xi )\rangle \mathop {}\!\textrm{d}V(x,\xi ) - \frac{c_0}{2\sigma ^{2}}\int \limits _{A_\sigma }\langle x-x_0, \star \xi \rangle \mathop {}\!\textrm{d}V(x,\xi ) \nonumber \\&\quad \quad + \frac{1}{2\rho ^2}\int \limits _{A_\rho }\langle x-x_0,H(x)-c_0(\star \xi )\rangle \mathop {}\!\textrm{d}V(x,\xi ) + \frac{c_0}{2\rho ^{2}}\int \limits _{A_\rho }\langle x-x_0, \star \xi \rangle \mathop {}\!\textrm{d}V(x,\xi ) \nonumber \\&\quad \quad +\frac{1}{2\sigma ^2}\int \limits _{B_\sigma }\langle x-x_0,\eta (x)\rangle \mathop {}\!\textrm{d}\beta _V(x) + \frac{1}{2}\int \limits _{B_\rho {\setminus } B_\sigma }\frac{\langle x-x_0,\eta (x)\rangle }{|x-x_0|^2}\mathop {}\!\textrm{d}\beta _V(x) \nonumber \\&\quad \quad - \frac{1}{2\rho ^2}\int \limits _{B_\rho }\langle x-x_0,\eta (x)\rangle \mathop {}\!\textrm{d}\beta _V(x). \end{aligned}$$

#### Proof of Lemma 4.1

Following the computations in [43, p. 284], we consider the smooth vector field $X(x):=x-x_0$ for $x\in {\mathbb {R}}^3$ and the Lipschitz function

4.2 $$\begin{aligned} \varphi :{\mathbb {R}}\rightarrow {\mathbb {R}},\qquad \varphi (t):=(\max \{t,\sigma \}^{-2} - \rho ^{-2})_+. \end{aligned}$$

Choose a sequence $\varphi _k$ in $C^\infty _c(-\infty ,\rho +1)$ such that $\sup _{k\in {\mathbb {N}}}\Vert \varphi _k\Vert _{C^1({\mathbb {R}})}<\infty$,

$$\begin{aligned}&\varphi _k \rightarrow \varphi{} & {} \hbox { locally uniformly as}\ k\rightarrow \infty ,\\&\varphi _k'(t) \rightarrow \varphi '(t){} & {} \text {as }k\rightarrow \infty \text { for all }t\in {\mathbb {R}}{\setminus }\{\sigma ,\rho \} \end{aligned}$$

and such that for all $k\in {\mathbb {N}}$, there holds $\varphi '_k(\sigma ) = 0$ and $\varphi '_k(\rho ) =- 2\rho ^{-3}$. Abbreviating $\Phi _k:=\varphi _k\circ |X|$ it follows

$$\begin{aligned} \lim _{k\rightarrow \infty }{{\,\textrm{div}\,}}_{T(\xi )}(\Phi _kX)(x) = {\left\{ \begin{array}{ll} 2(\frac{1}{\sigma ^2} - \frac{1}{\rho ^2}) &{} \hbox {for}\ (x,\xi )\in A_\sigma \\ \frac{2\langle X(x),\star \xi \rangle ^2}{|X(x)|^4} - \frac{2}{\rho ^2} &{} \hbox {for}\ (x,\xi )\in A_\rho {\setminus } A_\sigma \\ 0 &{} \text {for }(x,\xi )\in {\mathbb {G}}_2^\textrm{o}({\mathbb {R}}^3){\setminus } A_\rho . \end{array}\right. } \end{aligned}$$

Denoting $|X|_\sigma :=\max \{|X|,\sigma \}$, testing the first variation identity (see (2.2), and (2.1)) with the vector fields $\Phi _kX$ and passing to the limit as $k\rightarrow \infty$, we obtain

$$\begin{aligned}&\frac{2\mu _V(B_\sigma )}{\sigma ^2} + \int \limits _{A_{\rho }{\setminus } A_\sigma }\frac{2\langle X(x),\star \xi \rangle ^2}{|X(x)|^4}\mathop {}\!\textrm{d}V(x,\xi ) \\&\quad = \frac{2\mu _V(B_\rho )}{\rho ^2} - \int \limits _{B_\rho } (|X|_\sigma ^{-2} - \rho ^{-2})\langle X, H\rangle \mathop {}\!\textrm{d}\mu _V + \int \limits _{B_\rho } (|X|_\sigma ^{-2} - \rho ^{-2})\langle X, \eta \rangle \mathop {}\!\textrm{d}\beta _V. \end{aligned}$$

By (2.3) and since $|\star \xi |=1$ for $\xi \in {\mathbb {G}}^\textrm{o}(3,2)$, we have the pointwise identity

$$\begin{aligned} \left| \frac{1}{4}(H - c_0(\star \xi )) + \frac{\langle X,\star \xi \rangle (\star \xi )}{|X|^2}\right| ^2 = \frac{1}{16}|H-c_0(\star \xi )|^2 + \frac{\langle H - c_0(\star \xi ),X\rangle }{2|X|^2} + \frac{\langle X,\star \xi \rangle ^2}{|X|^4} \end{aligned}$$

and consequently

4.3 $$\begin{aligned}&\frac{\mu _V(B_\sigma )}{\sigma ^2} + \int \limits _{A_{\rho }{\setminus } A_\sigma } \left( \frac{1}{4}(\langle H(x),\star \xi \rangle - c_0) + \frac{\langle X(x), \star \xi \rangle }{|X(x)|^2}\right) ^2\mathop {}\!\textrm{d}V(x,\xi ) \nonumber \\&\quad = \frac{1}{16}\int \limits _{A_{\rho }{\setminus } A_\sigma }|H(x)-c_0(\star \xi )|^2\mathop {}\!\textrm{d}V(x,\xi ) + \frac{\mu _V(B_\rho )}{\rho ^2} \nonumber \\&\quad \quad + \frac{1}{2}\int \limits _{A_{\rho }{\setminus } A_\sigma } \frac{\langle H(x) - c_0(\star \xi ),X(x)\rangle }{|X(x)|^2}\mathop {}\!\textrm{d}V(x,\xi ) - \frac{1}{2}\int \limits _{B_\rho } (|X|_\sigma ^{-2} - \rho ^{-2})\langle X, H\rangle \mathop {}\!\textrm{d}\mu _V \nonumber \\&\quad \quad + \frac{1}{2} \int \limits _{B_\rho } (|X|_\sigma ^{-2} - \rho ^{-2})\langle X, \eta \rangle \mathop {}\!\textrm{d}\beta _V. \end{aligned}$$

Moreover, we have

4.4 $$\begin{aligned}&\frac{1}{2}\int \limits _{A_{\rho }{\setminus } A_\sigma } \frac{\langle H(x) - c_0(\star \xi ),X(x)\rangle }{|X(x)|^2}\mathop {}\!\textrm{d}V(x,\xi ) - \frac{1}{2}\int \limits _{B_\rho } (|X|_\sigma ^{-2} - \rho ^{-2})\langle X, H\rangle \mathop {}\!\textrm{d}\mu _V \nonumber \\&\quad = - \frac{c_0}{2}\int \limits _{A_{\rho }{\setminus } A_\sigma } \frac{\langle X(x), \star \xi \rangle }{|X(x)|^2}\mathop {}\!\textrm{d}V(x,\xi ) - \frac{1}{2\sigma ^2}\int \limits _{B_\sigma } \langle X, H\rangle \mathop {}\!\textrm{d}\mu _V + \frac{1}{2\rho ^2}\int \limits _{B_\rho } \langle X, H\rangle \mathop {}\!\textrm{d}\mu _V \nonumber \\&\quad = - \frac{c_0}{2}\int \limits _{A_{\rho }{\setminus } A_\sigma } \frac{\langle X(x), \star \xi \rangle }{|X(x)|^2}\mathop {}\!\textrm{d}V(x,\xi ) \nonumber \\&\quad \quad - \frac{1}{2\sigma ^2}\int \limits _{A_\sigma } \langle X(x), H(x) - c_0(\star \xi )\rangle \mathop {}\!\textrm{d}V(x,\xi ) - \frac{c_0}{2\sigma ^2}\int \limits _{A_\sigma } \langle X(x), \star \xi \rangle \mathop {}\!\textrm{d}V(x,\xi ) \nonumber \\&\quad \quad + \frac{1}{2\rho ^2}\int \limits _{A_\rho } \langle X(x), H(x) - c_0(\star \xi )\rangle \mathop {}\!\textrm{d}V(x,\xi )+ \frac{c_0}{2\rho ^2}\int \limits _{A_\rho } \langle X(x), \star \xi \rangle \mathop {}\!\textrm{d}V(x,\xi ) \end{aligned}$$

as well as

4.5 $$\begin{aligned}&\frac{1}{2} \int \limits _{B_\rho } (|X|_\sigma ^{-2} - \rho ^{-2})\langle X, \eta \rangle \mathop {}\!\textrm{d}\beta _V \nonumber \\&\quad = \frac{1}{2\sigma ^2}\int \limits _{B_\sigma }\langle X,\eta \rangle \mathop {}\!\textrm{d}\beta _V + \frac{1}{2}\int \limits _{B_\rho {\setminus } B_\sigma }\frac{\langle X,\eta \rangle }{|X|^2}\mathop {}\!\textrm{d}\beta _V - \frac{1}{2\rho ^2}\int \limits _{B_\rho }\langle X,\eta \rangle \mathop {}\!\textrm{d}\beta _V. \end{aligned}$$

Now, using $X(x) = x-x_0$ and putting (4.4), (4.5) into (4.3), the conclusion follows. $\square$

### The general varifold case

We now use the monotonicity formula (4.1) to prove our most general Li–Yau inequality.

#### Theorem 4.2

Suppose $V\in {\mathbb {V}}_2^{\textrm{o}}({\mathbb {R}}^3)$, $\eta \in L^\infty (\beta _V;{\mathbb {S}}^2)$ and $H\in L^1_\textrm{loc}(\mu _V;{\mathbb {R}}^3)$ satisfy Hypothesis 2.1. Let $c_0\in {\mathbb {R}}$ and suppose that

4.6 $$\begin{aligned} {\mathcal {H}}_{c_0}(V)<\infty \end{aligned}$$

and

4.7 $$\begin{aligned} \theta ^{*2}(\mu _V,\infty ):=\limsup _{\rho \rightarrow \infty }\frac{\mu _V(B_\rho (0))}{\pi \rho ^2}<\infty . \end{aligned}$$

Then, for all $x_0\in {\mathbb {R}}^3{\setminus } {{\,\textrm{spt}\,}}\beta _V$ we have

4.8 $$\begin{aligned} \theta ^2(\mu _V, x_0)&\le \theta ^{*2}(\mu _V,\infty ) + \frac{1}{4\pi }{\mathcal {H}}_{c_0}(V) \nonumber \\&\quad + \limsup _{\rho \rightarrow \infty }\frac{c_0}{2\pi }\left( \int \limits _{B_\rho (x_0)\times {\mathbb {G}}^{\textrm{o}}_2({\mathbb {R}}^3)}\left( \rho ^{-2}-|x-x_0|^{-2}\right) \langle x-x_0, \star \xi \rangle \mathop {}\!\textrm{d}V(x,\xi )\right) \nonumber \\&\quad + \limsup _{\rho \rightarrow \infty }\frac{1}{2\pi }\left( \int \limits _{B_\rho (x_0)}(|x-x_0|^{-2}-\rho ^{-2})\langle x-x_0, \eta (x)\rangle \mathop {}\!\textrm{d}\beta _V(x)\right) . \end{aligned}$$

#### Remark 4.3

(i) We do not assume $\mu _V({\mathbb {R}}^3)<\infty$ in Theorem 4.2. Indeed, let $r=1/c_0$ for $c_0>0$, let $f:{\mathbb {R}}\times {\mathbb {S}}^1\rightarrow {\mathbb {R}}^3$, $f(t, \varphi )=(r\cos \varphi , r\sin \varphi ,t)$ be the cylinder with radius *r*, let *V* be the associated varifold, cf. Example 2.4, and let $x_0=(r,0,0)\in {{\,\textrm{spt}\,}}\mu _V$. It is not difficult to see that $\beta =0$, ${\mathcal {H}}_{c_0}(V)=0$ and $\mu _V(B_\rho (x_0))=O(\rho )$ as $\rho \rightarrow \infty$, so that $\theta ^{*2}(\mu _V,\infty )=0$ whereas $\mu _V({\mathbb {R}}^3)=\infty$. Moreover, the third term on the right hand side of (4.8) is
  $$\begin{aligned} -\frac{c_0}{2\pi } \int \limits _{{\mathbb {R}}\times {\mathbb {S}}^1}\frac{\langle f-x_0, n\rangle }{|f-x_0|^2}\mathop {}\!\textrm{d}\mu&= \frac{c_0}{2\pi } \int \limits _0^{2\pi }\int \limits _{{\mathbb {R}}} \frac{a(\varphi )}{2a(\varphi ) +t^2}\mathop {}\!\textrm{d}t\mathop {}\!\textrm{d}\varphi , \end{aligned}$$
  where $a(\varphi ):=r^2(1-\cos \varphi )\ge 0$. Hence, the inner integral can be evaluated using the $\arctan$, yielding
  $$\begin{aligned} -\frac{c_0}{2\pi } \int \limits _{{\mathbb {R}}\times {\mathbb {S}}^1}\frac{\langle f-x_0, n\rangle }{|f-x_0|^2}\mathop {}\!\textrm{d}\mu&= \frac{c_0}{2\pi } \int \limits _0^{2\pi } \pi \sqrt{\frac{a(\varphi )}{2}} \mathop {}\!\textrm{d}\varphi = \frac{c_0}{2\pi }\cdot 4\pi r. \end{aligned}$$
  In the last step, we used $1-\cos \varphi = 2\sin ^2(\frac{\varphi }{2})$ and the symmetry of the sine function.
(ii) We can reverse the orientation of the varifold *V* by considering ${\hat{V}}$, the push forwad under the map $(x, \xi )\mapsto (x,-\xi )$, which is continuous and proper so ${\hat{V}}\in {\mathbb {V}}^{\textrm{o}}_2({\mathbb {R}}^3)$ by [18, 2.2.17]. In view of (1.1) it is not suprising that
  $$\begin{aligned} {\mathcal {H}}_{c_0}(V) = {\mathcal {H}}_{-c_0}({\hat{V}}). \end{aligned}$$
  Similarly, the other term in (4.8) involving $c_0$ remains unchanged if we replace *V* by ${\hat{V}}$ and $c_0$ by $-c_0$. The singular part does not change under reversing the orientation.
(iii) Equality holds for $c_0=0$ if *V* corresponds to the unit sphere and $x_0$ is any point on the unit sphere. Equality also holds for $c_0=0$ if *V* corresponds to the unit disk and $x_0$ is the center, and if *V* corresponds to a plane and $x_0$ is any point on the plane.
(iv) If the singular part $\beta _V$ is regular enough, for instance if ${{\,\textrm{spt}\,}}\beta _V$ is given by a smooth embedding $\gamma :{\mathbb {S}}^1\rightarrow {\mathbb {R}}^3$ and $\eta \circ \gamma$ is a unit normal field along $\gamma$, then the statement remains valid even for $x_0\in {{\,\textrm{spt}\,}}\beta _V$. Indeed, for *x* close to $x_0$, the vectors $x-x_0$ and $\eta (x)$ are nearly orthogonal. Thus, since $\theta ^1(\beta _V,x_0)=1$, a short argument using the Taylor expansion of $\gamma$ implies
  $$\begin{aligned} x\mapsto |x-x_0|^{-2}\langle x-x_0, \eta (x)\rangle \in L^1_{\textrm{loc}}(\beta _V). \end{aligned}$$

#### Proof of Theorem 4.2

For $\rho >0$ let $B_\rho$ and $A_\rho$ be as in Lemma 4.1. By Remark 3.3(i), there exist $D<\infty$ and $\rho _0>0$ such that

4.9 $$\begin{aligned} \mu (B_\rho )\le D\rho ^2 \quad \text {for all }0<\rho <\rho _0. \end{aligned}$$

Consequently, Lemma 3.2 yields

4.10 $$\begin{aligned} \int \limits _{B_{\rho }}\frac{1}{|x-x_0|}\mathop {}\!\textrm{d}\mu _V(x)\le C \rho \quad \text {for all }0<\rho < \rho _0, \end{aligned}$$

and thus $x\mapsto |x-x_0|^{-1}\in L^1_{\textrm{loc}}(\mu _V)$. Moreover we have $\textrm{dist}(x_0,{{\,\textrm{spt}\,}}\beta _V)>0$, and consequently

4.11 $$\begin{aligned} x\mapsto |x-x_0|^{-2}\langle x-x_0, \eta (x)\rangle \in L^1_{\textrm{loc}}(\beta _V). \end{aligned}$$

Using (4.6), (4.10) and (4.11), we find that the function $\gamma :(0,\infty )\rightarrow {\mathbb {R}}$ with

4.12 $$\begin{aligned} \gamma (\rho )&:=\frac{\mu _V(B_\rho )}{\rho ^2} + \frac{1}{16} \int \limits _{A_\rho }|H(x)-c_0(\star \xi )|^2\mathop {}\!\textrm{d}V(x,\xi ) - \frac{c_0}{2}\int \limits _{A_\rho }\frac{\langle x-x_0, \star \xi \rangle }{|x-x_0|^2}\mathop {}\!\textrm{d}V(x,\xi )\nonumber \\&\quad + \frac{1}{2\rho ^2}\int \limits _{A_\rho }\langle x-x_0, H(x)-c_0(\star \xi )\rangle \mathop {}\!\textrm{d}V(x,\xi ) + \frac{c_0}{2\rho ^2}\int \limits _{A_\rho }\langle x-x_0, \star \xi \rangle \mathop {}\!\textrm{d}V(x,\xi ) \nonumber \\&\quad + \frac{1}{2} \int \limits _{B_\rho }(|x-x_0|^{-2}-\rho ^{-2})\langle x-x_0, \eta (x)\rangle \mathop {}\!\textrm{d}\beta _V(x) \end{aligned}$$

is well defined and, by Lemma 4.1, it is monotonically nondecreasing.

We now examine the limit $\lim _{\sigma \rightarrow 0+}\gamma (\sigma )$. By (4.6), the second term in $\gamma (\sigma )$ goes to zero as $\sigma \rightarrow 0+$ and so does the third term by (4.10). For the fourth term, we use the Cauchy–Schwarz inequality to estimate

4.13 $$\begin{aligned}&\left|\sigma ^{-2}\int \limits _{A_\sigma }\langle x-x_0, H(x)-c_0(\star \xi )\rangle \mathop {}\!\textrm{d}V(x,\xi )\right| \nonumber \\&\qquad \le \left( \sigma ^{-2}\mu _V(B_\sigma )\right) ^{\frac{1}{2}}\left( \int \limits _{A_\sigma }|H(x)-c_0(\star \xi )|^2\mathop {}\!\textrm{d}V(x,\xi )\right) ^{\frac{1}{2}}, \end{aligned}$$

where the right hand side goes to zero by (4.6), (4.9) and since $\mu _V(\{x_0\})=0$ by Remark 3.3(i). The fifth term in $\gamma (\sigma )$ also goes to zero as $\sigma \rightarrow 0+$, since

4.14 $$\begin{aligned} \sigma ^{-2}\left|\int \limits _{A_\sigma } \langle x-x_0, \star \xi \rangle \mathop {}\!\textrm{d}V(x,\xi )\right|\le \sigma ^{-1}\mu _V(B_\sigma ) \le D\sigma , \end{aligned}$$

using (4.9). Since $x_0\not \in {{\,\textrm{spt}\,}}\beta _V$, we have $\beta _V(B_\sigma )=0$ for $\sigma >0$ sufficiently small. Consequently, using $\omega _2=\pi$, we find $\lim _{\sigma \rightarrow 0+}\gamma (\sigma )= \pi \theta ^{2}(\mu _V, x_0)$.

Now, we discuss the limit $\lim _{\rho \rightarrow \infty }\gamma (\rho )$. It is not too difficult to see that

4.15 $$\begin{aligned} \limsup _{\rho \rightarrow \infty }\frac{\mu _V(B_\rho )}{\pi \rho ^2} = \limsup _{\rho \rightarrow \infty }\frac{\mu _V(B_\rho (0))}{\pi \rho ^2} = \theta ^{*2}(\mu _V, \infty ). \end{aligned}$$

For the fourth term in (4.12), for any $0<\sigma <\rho$, we estimate by Cauchy–Schwarz

$$\begin{aligned}&\left|\rho ^{-2}\int \limits _{A_\rho }\langle x-x_0, H(x)-c_0(\star \xi )\rangle \mathop {}\!\textrm{d}V(x,\xi )\right|\\&\qquad \le \left( \rho ^{-2}\mu _V(B_\rho )\right) ^{\frac{1}{2}}\left( \int \limits _{{\mathbb {G}}_2^{\textrm{o}}({\mathbb {R}}^3){\setminus } A_\sigma } |H(x)-c_0(\star \xi )|^2\mathop {}\!\textrm{d}V(x,\xi )\right) ^{\frac{1}{2}} \\&\qquad \quad + \rho ^{-1}\int \limits _{A_\sigma }|H(x)-c_0(\star \xi )|\mathop {}\!\textrm{d}V(x,\xi ). \end{aligned}$$

Sending first $\rho \rightarrow \infty$ and then $\sigma \rightarrow \infty$, this goes to zero by (4.6), (4.7) and (4.15). The claim then follows from the monotonicity of $\gamma$. $\square$

If the singular part $\beta _V$ vanishes and ${{\,\mathrm{{\mathcal {V}}}\,}}_c(V,x_0)$ exists, using Lemma 3.2 we obtain the following

#### Corollary 4.4

Suppose $V\in {\mathbb {V}}^{\textrm{o}}_2({\mathbb {R}}^3)$ and $H\in L^2_\textrm{loc}(\mu _V;{\mathbb {R}}^3)$ satisfy Hypothesis 2.2. Let $c_0\in {\mathbb {R}}$, $x_0\in {\mathbb {R}}^3$ and suppose that ${{\,\mathrm{{\mathcal {V}}}\,}}_c(V,x_0)$ exists. Then we have

$$\begin{aligned} \theta ^{2}(\mu _V, x_0)\le \theta ^{* 2}(\mu _V,\infty )+ \frac{1}{4\pi }{\mathcal {H}}_{c_0}(V) + \frac{c_0}{2\pi } {{\,\mathrm{{\mathcal {V}}}\,}}_c(V,x_0). \end{aligned}$$

#### Proof

Without loss of generality, we may assume ${\mathcal {H}}_{c_0}(V)<\infty$, $\theta ^{* 2}(\mu _V,\infty )<\infty$. By Theorem 4.2, we only need to discuss the third term on the right hand side of (4.8). To that end, for $0<\sigma <\rho$ we estimate

$$\begin{aligned}&\frac{1}{\rho ^2}\int \limits _{A_\rho }|\langle x-x_0, \star \xi \rangle |\mathop {}\!\textrm{d}V(x,\xi ) \\&\qquad \le \int \limits _{{\mathbb {G}}^{\textrm{o}}_2({\mathbb {R}}^3){\setminus } A_\sigma } \frac{|\langle x-x_0, \star \xi \rangle |}{|x-x_0|^2}\mathop {}\!\textrm{d}V(x,\xi ) + \frac{1}{\rho ^2} \int \limits _{A_\sigma }|\langle x-x_0, \star \xi \rangle |\mathop {}\!\textrm{d}V(x,\xi ). \end{aligned}$$

Sending first $\rho \rightarrow \infty$ and then $\sigma \rightarrow \infty$ this goes to zero since ${{\,\mathrm{{\mathcal {V}}}\,}}_c(V,x_0)$ exists by assumption. The result follows. $\square$

### Varifolds with enclosed volume

In this section we introduce a class of oriented varifolds that satisfy a divergence theorem, see Hypothesis 4.5. These varifolds comprise the surfaces shown in Fig. 2a–e, h. We then show that their concentrated volume is positive, see Lemma 4.9. We start with a short review of sets of locally finite perimeter, cf. [17, Chapter 5], [18, Section 4.5].

Let $E\subset {\mathbb {R}}^3$. We define the *measure theoretic boundary of* *E* by

$$\begin{aligned} \partial _*E = \{x\in {\mathbb {R}}^3 \mid \theta ^{*3}({\mathcal {L}}^3\llcorner E,x)>0,\,\theta ^{*3}({\mathcal {L}}^3\llcorner ({\mathbb {R}}^3{\setminus } E),x)>0\}. \end{aligned}$$

Moreover, we denote with $n_E:{\mathbb {R}}^3\rightarrow {\mathbb {R}}^3$ the *measure theoretic inner unit normal of* *E* (see the definition [18, 4.5.5]). In view of *Federer’s criterion* [18, 4.5.11, 2.10.6], we say that *E* has *locally finite perimeter*, if and only if *E* is an ${\mathcal {L}}^3$-measurable set, and ${\mathcal {H}}^2(K\cap \partial _*E)<\infty$ for all compact sets $K\subset {\mathbb {R}}^3$.

Let $E\subset {\mathbb {R}}^3$ be a set of locally finite perimeter and $B = \{x\in {\mathbb {R}}^3\mid |n_E(x)|=1\}$. We collect the following properties (see [18, 4.5.6]).

- The sets *B* and $\partial _*E$ are ${\mathcal {H}}^2$-almost equal.
- ${\mathcal {H}}^2\llcorner \partial _*E$ is a Radon measure over ${\mathbb {R}}^3$ and $n_E$ is ${\mathcal {H}}^2\llcorner \partial _*E$-measurable.
- The divergence theorem reads as
  4.16 $$\begin{aligned} -\int \limits _{\partial _*E}\langle X,n_E\rangle \mathop {}\!\textrm{d}{\mathcal {H}}^2 = \int \limits _E{{\,\textrm{div}\,}}X\mathop {}\!\textrm{d}{\mathcal {L}}^3 \end{aligned}$$
  for all Lipschitz maps $X:{\mathbb {R}}^3\rightarrow {\mathbb {R}}^3$ with compact support.

In view of Riesz’s representation theorem, we define the oriented varifold $V\in {\mathbb {V}}_2^\textrm{o}({\mathbb {R}}^3)$ associated with $\partial _*E$ by

4.17 $$\begin{aligned} V(\varphi ) :=\int \limits _{{\mathbb {R}}^3}\varphi (x,\star n_E(x))\,\mathrm d({\mathcal {H}}^2\llcorner \partial _*E)(x) \end{aligned}$$

for all real valued continuous functions $\varphi$ on $\mathbb G^\textrm{o}_2({\mathbb {R}}^3)$ with compact support. There holds $\mu _V = {\mathcal {H}}^2\llcorner \partial _*E$ and (4.16) reads

$$\begin{aligned} \int \limits _{{\mathbb {G}}_2^\textrm{o}({\mathbb {R}}^3)}\langle X(x),\star \xi \rangle \mathop {}\!\textrm{d}V(x,\xi ) = - \int \limits _{E}{{\,\textrm{div}\,}}X\mathop {}\!\textrm{d}{\mathcal {L}}^3 \end{aligned}$$

for all Lipschitz maps $X:{\mathbb {R}}^3\rightarrow {\mathbb {R}}^3$ with compact support.

This divergence theorem is the main motivation for considering a particular class of varifolds in the sequel.

#### Hypothesis 4.5

Suppose $V\in {\mathbb {V}}_2^\textrm{o}({\mathbb {R}}^3)$ and $H\in L_\textrm{loc}^1(\mu _V;{\mathbb {R}}^3)$ satisfy Hypothesis 2.2, $E\subset {\mathbb {R}}^3$ is an ${\mathcal {L}}^3$-measurable set, $\Theta \in L^1_{\textrm{loc}}({\mathcal {L}}^3\llcorner E;{\mathbb {N}})$,

4.18 $$\begin{aligned} {{\,\textrm{diam}\,}}{{\,\textrm{spt}\,}}({\mathcal {L}}^3\llcorner E) \le {{\,\textrm{diam}\,}}{{\,\textrm{spt}\,}}\mu _V, \end{aligned}$$

and

4.19 $$\begin{aligned} -\int \limits _{{\mathbb {G}}_2^\textrm{o}({\mathbb {R}}^3)}\langle X(x),\star \xi \rangle \mathop {}\!\textrm{d}V(x,\xi ) = \int \limits _{E}({{\,\textrm{div}\,}}X) \Theta \mathop {}\!\textrm{d}{\mathcal {L}}^3 \end{aligned}$$

for all Lipschitz maps $X:{\mathbb {R}}^3\rightarrow {\mathbb {R}}^3$ with compact support. In this case, we term *V* *a varifold with enclosed volume*.

#### Remark 4.6

(i) If $V\in {\mathbb {V}}^{\textrm{o}}_2({\mathbb {R}}^3)$ is integral with compact support and such that the associated 2-current has zero boundary, by [18, 4.5.17] we find that (4.19) is satisfied for some measurable $E\subset {\mathbb {R}}^3$ and $\Theta \in L^1_{\textrm{loc}}({\mathcal {L}}^3\llcorner E;{\mathbb {Z}})$, see also [15, Section 3]. In Hypothesis 4.5 we additionally require $\Theta >0$ a.e. on *E*, the diameter bound (4.18) and that *V* satisfies Hypothesis 2.2.
(ii) The sign in Eq. (4.19) is adapted to our convention that the unit normal points to the interior.
(iii) Since the divergence theorem (4.16) remains true if we replace *E* with ${\mathbb {R}}^3{\setminus } E$ and $n_E$ with $-n_E$, condition (4.18) ensures that we pick the correct orientation.
(iv) The function $\Theta$ has *locally bounded variation* (see the definition [17, Section 5.1]) and the coarea formula [18, 4.5.9(13)] implies that
  $$\begin{aligned} E_k:=\{x\in {\mathbb {R}}^3\mid \Theta (x) \ge k\} \qquad \text {for } k\in {\mathbb {N}}\end{aligned}$$
  defines a sequence of decreasing sets of locally finite perimeter.
(v) If $\Theta \equiv 1$, then the varifold associated with $\partial _*E$ does not necessarily coincide with *V*, compare Fig. 2e, f.
(vi) If *V* is associated with the reduced boundary of a set *E* of locally finite perimeter, then $q_\#V$ is an integral varifold (in the sense of [2, 3.5]). Hence, if additionally *V* has generalized mean curvature *H* and vanishing singular part $\beta _V = 0$, then there holds $H(x)\wedge \star \xi = 0$ for *V*-almost all $(x,\xi )$ by [7, Section 5.8], *V*, *H* satisfy Hypothesis 2.2 and thus *V*, *H*, *E* and $\Theta \equiv 1$ satisfy Hypothesis 4.5.

As the following example shows, not all varifolds associated with sets of finite perimeter satisfy Hypothesis 4.5.

#### Example 4.7

Let $C_\alpha$ be the closed spherical cap of the unit sphere with opening angle $\alpha = \pi /3$ (the hemisphere has opening angle $\pi /2$) whose boundary circle lies in the plane $\{z=0\}$. Let $S = C_\alpha \cup (-C_\alpha )$, i.e. *S* is the gluing of the spherical cap $C_\alpha$ with its reflection at the plane $\{z=0\}$. The surface *S* looks like a lens, see Fig. 2g. Its singular part is the circle $\Gamma _a$ of radius $a=\sqrt{3}/2$ centered at the origin and lying in the plane $\{z=0\}$. Since ${\mathcal {H}}^2(S)<4\pi <\infty$, one can use Federer’s criterion to show that *S* is the boundary of a set *E* of finite perimeter. However, the varifold *V* associated with $S=\partial _*E$ (cf. (4.17)) does not satisfy Hypothesis 4.5. In fact, *V* does not satisfy Hypothesis 2.2, but the more general Hypothesis 2.1.

Indeed, in view of [2, 4.4, 4.7], there holds $\mu _V = {\mathcal {H}}^2\llcorner S$, $\beta _V = \sqrt{3}{\mathcal {H}}^1\llcorner \Gamma _a$, and

$$\begin{aligned} \delta V(X) = -\int \limits _{S}\langle X,H\rangle \mathop {}\!\textrm{d}{\mathcal {H}}^2 + \sqrt{3}\int \limits _{\Gamma _a}\frac{\langle X(x),x\rangle }{|x|} \mathop {}\!\textrm{d}{\mathcal {H}}^1(x) \end{aligned}$$

where *H* is the mean curvature of the spherical caps $\pm C_\alpha$. Notice that $\theta ^1(\beta _V,x)=\sqrt{3}$ for all $x\in \Gamma _a$. In other words, $\beta _V$ does not have integer multiplicity even though $\theta ^2(\mu _V,x) = 1$ for all $x\in S$. Notice also that *V* satisfies the hypothesis of Theorem 4.2 for all $c_0\in {\mathbb {R}}$.

The set *E* in Hypothesis 4.5 corresponds to an enclosed volume in the following sense, where the algebraic volume does not depend on the point $x_0\in {\mathbb {R}}^3$.

#### Proposition 4.8

Suppose $V, H, E, \Theta$ satisfy Hypothesis 4.5 with ${{\,\textrm{spt}\,}}\mu _V$ compact. Then

$$\begin{aligned} {{\,\mathrm{{\mathcal {V}}}\,}}(V, x_0)= \int \limits _{E}\Theta \mathop {}\!\textrm{d}{\mathcal {L}}^3 =:{{\,\mathrm{{\mathcal {V}}}\,}}(V)\qquad \text { for all }x_0\in {\mathbb {R}}^3. \end{aligned}$$

#### Proof

Since ${{\,\textrm{spt}\,}}\mu _V$ is compact, so is ${{\,\textrm{spt}\,}}({\mathcal {L}}^3\llcorner E)$ by Hypothesis 4.5. We may thus apply (4.19) with $X(x)=x-x_0$, suitably cutoff away from ${{\,\textrm{spt}\,}}\mu _V$ and ${{\,\textrm{spt}\,}}({\mathcal {L}}^3\llcorner E)$. $\square$

Under suitable assumptions, the concentrated volume can be computed by (4.19), too.

#### Lemma 4.9

Suppose $V,H,E,\Theta$ satisfy Hypothesis 4.5. Let $x_0\in {\mathbb {R}}^3$ and assume

4.20 $$\begin{aligned} \lim _{\rho \rightarrow \infty }\frac{1}{\rho ^2}\int \limits _{E\cap B_\rho (x_0)}\Theta \mathop {}\!\textrm{d}{\mathcal {L}}^3 =0. \end{aligned}$$

Then we have

4.21 $$\begin{aligned} {{\,\mathrm{{\mathcal {V}}}\,}}_c(V,x_0) = \int \limits _E\frac{\Theta (x)}{|x-x_0|^2}\mathop {}\!\textrm{d}{\mathcal {L}}^3(x), \end{aligned}$$

provided both sides exist.

#### Remark 4.10

(i) By Proposition 3.4, if $V\in {\mathbb {V}}^{\textrm{o}}_2({\mathbb {R}}^3)$ and $H\in L^2_{\textrm{loc}}(\mu _V;{\mathbb {R}}^3)$ satisfy Hypothesis 2.2 and if ${{\,\mathrm{{\mathcal {V}}}\,}}(V,x_0)$ exists, then also ${{\,\mathrm{{\mathcal {V}}}\,}}_c(V,x_0)$ exists.
(ii) Suppose $\int \limits _{E}\Theta \mathop {}\!\textrm{d}{\mathcal {L}}^3<\infty$. By Lemma 3.2 applied to the measure $\Theta {\mathcal {L}}^3\llcorner E$, the right hand side of (4.21) exists if for some $m>2$ we have
  4.22 $$\begin{aligned} \limsup _{\sigma \rightarrow 0+} \frac{1}{\sigma ^m}\int \limits _{E\cap B_\sigma (x_0)}\Theta \mathop {}\!\textrm{d}{\mathcal {L}}^3 <\infty . \end{aligned}$$
  As a consequence of the Lebesgue differentiation theorem, this is true for $m=3$ and ${\mathcal {L}}^3$-almost all $x_0\in E$ (cf. [18, 2.9.8]). However, not all $\Theta \in L^1_{\textrm{loc}}({\mathcal {L}}^3\llcorner E;{\mathbb {N}})$ and $x_0\in {\mathbb {R}}^3$ satisfy (4.22). This can be seen by taking $\Theta (x):=\lceil |x-x_0|^{-2}\rceil$. Nevertheless, (4.22) is clearly satisfied if $\Theta \in L^{\infty }({\mathcal {L}}^3\llcorner E;{\mathbb {N}})$.

#### Proof of Lemma 4.9

Since ${{\,\mathrm{{\mathcal {V}}}\,}}_c(V,x_0)$ exists, we have

4.23 $$\begin{aligned} \int \limits _{{\mathbb {G}}^{\textrm{o}}_2({\mathbb {R}}^3)}\frac{|\langle x-x_0, \star \xi \rangle |}{|x-x_0|^2}\mathop {}\!\textrm{d}V(x,\xi )<\infty . \end{aligned}$$

Now, let $0<\sigma <\rho$ and let $B_\rho , A_\rho$ be as in Lemma 4.1. Moreover, let $\varphi$ be as in (4.2), $X(x):=x-x_0$, $\Phi (x):=\varphi (|x-x_0|)$ for $x\in {\mathbb {R}}^3$. For ${\mathcal {L}}^3$-almost every $x\in {\mathbb {R}}^3$ we find

$$\begin{aligned} {{\,\textrm{div}\,}}\big (\Phi X\big )(x)&= \left\{ \begin{array}{ll} 3(\sigma ^{-2}-\rho ^{-2}) &{} \text { for } x\in B_\sigma \\ |X(x)|^{-2}- 3\rho ^{-2}&{} \text { for } x\in B_\rho {\setminus } B_\sigma \\ 0 &{} \text { for } x\in {\mathbb {R}}^3{\setminus } B_\rho . \end{array}\right. \end{aligned}$$

Thus (4.19) implies

4.24 $$\begin{aligned}&-\frac{1}{\sigma ^2}\int \limits _{A_\sigma }\langle X(x), \star \xi \rangle \mathop {}\!\textrm{d}V(x,\xi )+ \frac{1}{\rho ^2}\int \limits _{A_\rho }\langle X(x),\star \xi \rangle \mathop {}\!\textrm{d}V(x,\xi ) - \int \limits _{A_\rho {\setminus } A_\sigma }\frac{\langle X(x), \star \xi \rangle }{|X(x)|^2}\mathop {}\!\textrm{d}V(x,\xi ) \nonumber \\&\quad = \frac{3}{\sigma ^2}\int \limits _{E\cap B_\sigma }\Theta \mathop {}\!\textrm{d}{\mathcal {L}}^3- \frac{3}{\rho ^2}\int \limits _{E\cap B_\rho }\Theta \mathop {}\!\textrm{d}{\mathcal {L}}^3 + \int \limits _{E\cap B_\rho {\setminus } B_\sigma } \frac{\Theta (x)}{|X(x)|^2}\mathop {}\!\textrm{d}{\mathcal {L}}^3(x). \end{aligned}$$

We analyze each term in (4.24) separately. First, as $\sigma \rightarrow 0+$, the first term on the left vanishes, since (4.23) yields

$$\begin{aligned} \frac{1}{\sigma ^2}\int \limits _{A_\sigma }|\langle X(x), \star \xi \rangle |\mathop {}\!\textrm{d}V(x,\xi )\le \int \limits _{A_\sigma }\frac{|\langle X(x), \star \xi \rangle |}{|X(x)|^2}\mathop {}\!\textrm{d}V(x,\xi )\rightarrow 0. \end{aligned}$$

Here we used that $\mu _V(\{x_0\})=0$ by Remark 3.3(i). The first term on the right hand side of (4.24) goes to zero as $\sigma \rightarrow 0$, since the right hand side of (4.21) exists. For the second term on the left, taking $0<r<\rho$ and splitting the integral we obtain

$$\begin{aligned}&\frac{1}{\rho ^2}\int \limits _{A_\rho }|\langle X(x), \star \xi \rangle |\mathop {}\!\textrm{d}V(x,\xi ) \\&\qquad \le \int \limits _{{\mathbb {G}}^{\textrm{o}}_2({\mathbb {R}}^3){\setminus } A_r} \frac{|\langle X(x), \star \xi \rangle |}{|X(x)|^2}\mathop {}\!\textrm{d}V(x,\xi ) + \frac{1}{\rho ^2} \int \limits _{A_r}|\langle X(x), \star \xi \rangle |\mathop {}\!\textrm{d}V(x,\xi ), \end{aligned}$$

which goes to zero by (4.23), if we send first $\rho \rightarrow \infty$ and then $r\rightarrow \infty$. Taking $\rho \rightarrow \infty$ the second term on the right of (4.24) vanishes by (4.20). Thus, if we let first $\sigma \rightarrow 0$ and then $\rho \rightarrow \infty$ in (4.24) and use that both sides of (4.21) exist, the claim follows. $\square$

By the preceding discussion, the statement of Corollary 4.4 can be simplified if *V* is a varifold with enclosed volume. For simplicity, we only consider the case where ${{\,\textrm{spt}\,}}\mu _V$ is compact.

#### Corollary 4.11

Suppose $V,H,E, \Theta$ satisfy Hypothesis 4.5 with ${{\,\textrm{spt}\,}}\mu _V$ compact. Then

$$\begin{aligned} \theta ^2(\mu _V,x_0)\le {\mathcal {H}}_{c_0}(V) + \frac{c_0}{2\pi }\int \limits _{E}\frac{\Theta (x)}{|x-x_0|^2}\mathop {}\!\textrm{d}{\mathcal {L}}^3(x) \end{aligned}$$

for all $x_0\in {\mathbb {R}}^3$, provided the second term on the right hand side exists.

#### Proof

By Hypothesis 4.5 we find that ${{\,\textrm{spt}\,}}({\mathcal {L}}^3\llcorner E)$ is compact, so that using Remark 2.3 and Remark 4.10(i) we find that the assumptions of Corollary 4.4 and Lemma 4.9 are satisfied. The result then directly follows using (4.21). $\square$

## The smooth setting

In this section, we will transfer the general varifold Li–Yau inequalities to the setting of smoothly immersed surfaces.

### Proofs of the Li–Yau inequalities

Lemma 1.3 is an easy consequence of the varifold result.

#### Proof of Lemma 1.3

The claim follows directly from Corollary 4.4 if we consider the varifold associated to the immersion *f*, cf. Example 2.4. $\square$

We now show that any Alexandrov immersion induces a varifold with enclosed volume.

#### Lemma 5.1

Let $\Sigma$ be a closed surface and let $f:\Sigma \rightarrow {\mathbb {R}}^3$ be an Alexandrov immersion with $\Sigma =\partial M$, $f=F\vert _{\Sigma }$ and $F:M\rightarrow {\mathbb {R}}^3$ as in Definition 1.4. Let *V* be the oriented 2-varifold on ${\mathbb {R}}^3$ associated to $(\Sigma ,f)$ as in Example 2.4. Then, there holds

$$\begin{aligned} - \int \limits _{{\mathbb {G}}_2^\textrm{o}({\mathbb {R}}^3)} \langle X(x), \star \xi \rangle \mathop {}\!\textrm{d}V(x,\xi ) = \int \limits _{F[M]} ({{\,\textrm{div}\,}}X)(x) {\mathcal {H}}^{0}(F^{-1}\{x\}) \mathop {}\!\textrm{d}{\mathcal {L}}^3(x) \end{aligned}$$

for all Lipschitz $X:{\mathbb {R}}^3\rightarrow {\mathbb {R}}^3$ with compact support. In particular, with $E:=F[M]$, $\Theta :={\mathcal {H}}^{0}(F^{-1}\{\cdot \})$ we see that $V, H, E, \Theta$ satisfy Hypothesis 4.5.

#### Proof

By an approximation argument, it suffices to consider $X\in C_c^{1}({\mathbb {R}}^3;{\mathbb {R}}^3)$. Denote with $\Omega$ the Riemannian measure on *M* induced by the pullback metric $g_F:=F^*\langle \cdot ,\cdot \rangle$, let $\mu$ be the induced measure on $\Sigma$, and let $\nu$ be the inner unit normal on $\Sigma$. Given any vector field $X \in C^1({\mathbb {R}}^3;{\mathbb {R}}^3)$, we define the vector field $X^*$ on *M* by $X^*(p) = (\mathrm d F_p)^{-1}(X(F(p)))$. By (2.7) and since $n=\mathop {}\!\textrm{d}F(\nu )$, we compute

$$\begin{aligned} -\int \limits _{{\mathbb {G}}^\textrm{o}_2({\mathbb {R}}^3)} \langle X(x),\star \xi \rangle \mathop {}\!\textrm{d}V(x,\xi )&= - \int \limits _{\Sigma } \langle X\circ f, n \rangle \mathop {}\!\textrm{d}\mu = \int \limits _{\partial M}g_F(X^*,-\nu )\mathop {}\!\textrm{d}\mu . \end{aligned}$$

Since $(M,g_F)$ is flat, we have ${{\,\textrm{div}\,}}_{g_F} X^* = ({{\,\textrm{div}\,}}X) \circ F$. Hence, by the divergence theorem for Riemannian manifolds (see [36, Theorem 5.11(2)]) and the area formula,

$$\begin{aligned} \int \limits _{\partial M}g_F(X^*,-\nu )\mathop {}\!\textrm{d}\mu = \int \limits _M ({{\,\textrm{div}\,}}X)\circ F\mathop {}\!\textrm{d}\Omega = \int \limits _{F[M]}({{\,\textrm{div}\,}}X)(x){\mathcal {H}}^0(F^{-1}\{x\})\mathop {}\!\textrm{d}{\mathcal {L}}^3(x) \end{aligned}$$

which implies the conclusion. $\square$

Equipped with this tool we can now prove Theorem 1.5.

#### Proof of Theorem 1.5

By Lemma 5.1, $V, H, E:=F[M], \Theta :={\mathcal {H}}^{0}(F^{-1}\{\cdot \})$ satisfy Hypothesis 4.5. Since *M* is compact and *F* is a local diffeomorphism, there exists $k\in {\mathbb {N}}$ such that

$$\begin{aligned} \Theta (x)={\mathcal {H}}^{0}(F^{-1}\{x\}) \le k \quad \text {for all }x\in E=F[M], \end{aligned}$$

and as a consequence of Lemma 4.9 and Remark 4.10(i) and (ii) we find

5.1 $$\begin{aligned} {{\,\mathrm{{\mathcal {V}}}\,}}_c(f, x_0) = \int \limits _{F[M]}\frac{{\mathcal {H}}^{0}(F^{-1}\{x\})}{|x-x_0|^2}\mathop {}\!\textrm{d}{\mathcal {L}}^3(x) \quad \text {for all }x_0\in {\mathbb {R}}^3. \end{aligned}$$

The statement then follows from Corollary 4.11. $\square$

#### Remark 5.2

The results of Lemma 1.3 and Theorem 1.5 are sharp in the sense that equality can be achieved asymptotically for every $c_0\in {\mathbb {R}}$. Indeed, let ${\mathbb {S}}^2\subset {\mathbb {R}}^3$ be the unit sphere, and let $f:{\mathbb {S}}^2\rightarrow {\mathbb {R}}^3, f(x)=rx$ denote the parametrization of the round sphere $\partial B_r(0)\subset {\mathbb {R}}^3$ with radius $r>0$ and the orientation given by the inner unit normal. This is clearly an Alexandrov immersion (with $M=B_1(0)$, $F(x)=rx$) and hence by (5.1), we have

5.2 $$\begin{aligned} {{\,\mathrm{{\mathcal {V}}}\,}}_{c}(f,x_0)&= \int \limits _{B_r(0)}\frac{1}{|x-x_0|^2}\mathop {}\!\textrm{d}{\mathcal {L}}^3(x) = {\left\{ \begin{array}{ll} 2\pi r &{} \text { if }x_0\in \partial B_r(0),\\ 4\pi r &{} \text { if }x_0=0. \end{array}\right. } \end{aligned}$$

To verify the last equality in (5.2) we use the original surface integral definition (1.7) for the concentrated volume and that $n=-f/r$. The claim then follows from

$$\begin{aligned} {{\,\mathrm{{\mathcal {V}}}\,}}_c(f,x_0)= \frac{1}{r}\int \limits _{{\mathbb {S}}^2}\frac{\langle f-x_0,f\rangle }{|f-x_0|^2}\mathop {}\!\textrm{d}\mu = \frac{1}{r}\int \limits _{{\mathbb {S}}^2} \frac{r^2-\langle x_0, f\rangle }{r^2+|x_0|^2-2\langle f, x_0\rangle }\mathop {}\!\textrm{d}\mu . \end{aligned}$$

If now $x_0\in \partial B_r(0)$, Inequality (1.8) reads

$$\begin{aligned} 1={\mathcal {H}}^{0}(f^{-1}\{x_0\})\le \frac{1}{4\pi }{\mathcal {H}}_{c_0}(f) + \frac{c_0}{2\pi }{{\,\mathrm{{\mathcal {V}}}\,}}_c(f,x_0) = \frac{1}{4}\left( c_0r-2\right) ^2 + c_0 r \quad \text { for all }r>0, \end{aligned}$$

where the right hand side converges to 1 as $r\rightarrow 0+$. In the case $c_0=0$, equality is achieved by any round sphere.

### A scale-invariant version

Clearly, for $x_0=0$ the left hand sides of the Li–Yau inequalities in Lemma 1.3 and Theorem 1.5 are invariant under rescalings of the immersion, whereas the right hand sides are not. We will now prove a scale-invariant version of the inequality, involving the $L^2$-*CMC-deficit* of an immersion $f:\Sigma \rightarrow {\mathbb {R}}^3$ of an oriented surface $\Sigma$, given by

$$\begin{aligned} \bar{{\mathcal {H}}}(f) = \frac{1}{4}\int \limits _{\Sigma }(H_{\textrm{sc}}-\bar{H}_{\textrm{sc}})^2\mathop {}\!\textrm{d}\mu =\inf _{c_0\in {\mathbb {R}}}{\mathcal {H}}_{c_0}(f), \end{aligned}$$

cf. (1.3). Here ${\bar{H}}_{\textrm{sc}}:={\mathcal {A}}(f)^{-1} {\int \limits _{\Sigma } H_{\textrm{sc}}\mathop {}\!\textrm{d}\mu }$ denotes the *average scalar mean curvature*, provided the latter integral exists. Note that $\bar{{\mathcal {H}}}(f) = 0$ if and only if *f* is an immersion with constant mean curvature, a *CMC-immersion*, justifying the terminology. We obtain the following Li–Yau inequality which is invariant under rescaling and also under reversing the orientation on $\Sigma$.

#### Corollary 5.3

Let $f:\Sigma \rightarrow {\mathbb {R}}^3$ be an immersion of a compact oriented surface $\Sigma$ without boundary. Then for all $x_0\in {\mathbb {R}}^3$ we have

5.3 $$\begin{aligned} {\mathcal {H}}^{0}(f^{-1}\{x_0\})\le \frac{1}{4\pi } \bar{{\mathcal {H}}}(f) + \frac{1}{2\pi } {\bar{H}}_{\textrm{sc}} {{\,\mathrm{{\mathcal {V}}}\,}}_{c}(f,x_0) - \frac{1}{\pi {\mathcal {A}}(f)}\left( {{\,\mathrm{{\mathcal {V}}}\,}}_c(f,x_0)\right) ^2. \end{aligned}$$

#### Proof

By Proposition 3.4, we find that ${{\,\mathrm{{\mathcal {V}}}\,}}_c(f,x_0)$ exists. We may thus use Lemma 1.3 for any $c_0\in {\mathbb {R}}$. Expanding the right hand side of (1.8), we obtain a quadratic polynomial in $c_0$. By a direct computation, this polynomial is minimal for $c_0 =\frac{\int _\Sigma H_{\textrm{sc}}\mathop {}\!\textrm{d}\mu - 4 {{\,\mathrm{{\mathcal {V}}}\,}}_c(f,x_0)}{{\mathcal {A}}(f)}$ and the minimal value is precisely the right hand side of (5.3). $\square$

## Applications

In this section, we discuss several applications of the Li–Yau inequalities. We first provide a lower bound on the Helfrich energy resulting in nonexistence of minimizers for the penalized Canham–Helfrich model in Sect. 6.1. In Sect. 6.2 we prove some important geometric estimates involving the Helfrich energy. We then use these to prove Theorem 1.6. Lastly, we discuss a criterion for positive total mean curvature in Sect. 6.4.

### Nonexistence of minimizers for the penalized Canham–Helfrich model

#### Lemma 6.1

Suppose $V\in {\mathbb {V}}_2^\textrm{o}({\mathbb {R}}^3)$ and $H\in L_\textrm{loc}^2(\mu _V)$ satisfy Hypothesis 2.2, ${{\,\textrm{spt}\,}}\mu _V$ is compact, $c_0 < 0$, and $x_0\in {\mathbb {R}}^3$ such that $\theta ^{*2}(\mu _V,x_0) \ge 1$ and ${{\,\mathrm{{\mathcal {V}}}\,}}_c(V,x_0)>0$. Then there holds

$$\begin{aligned} {\mathcal {H}}_{c_0}(V) > 4\pi . \end{aligned}$$

#### Proof

This is a consequence of Corollary 4.4 in combination with [39, Theorem 3.6]. $\square$

#### Remark 6.2

The proof of the above inequality for the Willmore functional (i.e. $c_0=0$) [44, Theorem 7.2.2] also works for the Helfrich functional provided *V* is given by an Alexandrov immersion $f:\Sigma \rightarrow {\mathbb {R}}^3$ with inner unit normal field *n*. Indeed, denoting with $K^+$ the set of points in $\Sigma$ where both principal curvatures are nonnegative, we find

$$\begin{aligned} {\mathcal {H}}_{c_0}(f)&\ge \frac{1}{4}\int \limits _{K^+}|H_f - c_0n|^2\mathop {}\!\textrm{d}\mu \\&\ge \frac{1}{4}\int \limits _{K^+}|H_f|^2\mathop {}\!\textrm{d}\mu + \frac{c_0^2}{4}{\mathcal {A}}(f) > \frac{1}{4}\int \limits _{K^+}|H_f|^2\mathop {}\!\textrm{d}\mu \ge \int \limits _{K^+}K\mathop {}\!\textrm{d}\mu \end{aligned}$$

where *K* denotes the Gauss curvature. Similarly to [44, Lemma 7.2.1] we see that if *f* is an Alexandrov immersion, then

$$\begin{aligned} \int \limits _{K^+}K\mathop {}\!\textrm{d}\mu \ge 4\pi . \end{aligned}$$

For all real numbers $c_0,\lambda ,p$ we define the energy functional

$$\begin{aligned} {\mathcal {H}}^{\lambda ,p}_{c_0}(f) :={\mathcal {H}}_{c_0}(f) + \lambda {\mathcal {A}}(f) + p{{\,\mathrm{{\mathcal {V}}}\,}}(f) \end{aligned}$$

for all smooth immersions $f:\Sigma \rightarrow {\mathbb {R}}^3$ of a closed oriented surface $\Sigma$. The constants $\lambda$ and *p* are referred to as *tensile stress* and *osmotic pressure*. The energy was considered by Zhong-Can and Helfrich [45, Equation (1)] in the study of spherical vesicles. Each minimizer of the constrained minimization Problem 1.1 is a critical point of the functional ${\mathcal {H}}^{\lambda ,p}_{c_0}$ for some $\lambda ,p$ by the method of Lagrange multipliers. This is one of the reasons why the energy ${\mathcal {H}}^{\lambda ,p}_{c_0}$ is subject of numerous works in mathematical physics, biology and mathematics, see for instance [5] and the references therein.

Denote with ${\mathcal {S}}^\infty$ the set of smoothly embedded spheres in ${\mathbb {R}}^3$. In view of (1.2), we see that

6.1 $$\begin{aligned} \inf _{f\in {\mathcal {S}}^\infty }{\mathcal {H}}^{\lambda ,p}_{c_0}(f) \le 4\pi . \end{aligned}$$

In [37, Theorem 3] (see also [31, Theorem 1.9]) the existence of spheres minimizing ${\mathcal {H}}^{\lambda ,p}_{c_0}$ was shown, provided $\lambda ,c_0>0$ and $p\ge 0$. However, in view of [13], $c_0<0$ is empirically more relevant in the study of red blood cells. Lemma 6.1 now reveals that the infimum in (6.1) is not attained whenever $c_0<0$ and $\lambda ,p \ge 0$. This is actually in accordance with the results on the gradient flow in [6, 28]. Notice also the different behaviour of the constrained gradient flow [35]. Examining the scaling behaviour of ${\mathcal {H}}^{\lambda ,p}_{c_0}$ evaluated at round spheres, we see that the energy functional is unbounded from below if $p<0$; in particular, the infimum in (6.1) is not attained. Similarly, if $\lambda <0$ and $c_0^2+\lambda <0$, one can use surfaces of degenerating isoperimetric ratio found in [38, Theorem 1.5] to construct a sequence of embeddings $f_k$ in ${\mathcal {S}}^\infty$ such that ${\mathcal {H}}^{\lambda ,p}_{c_0}(f_k) \rightarrow -\infty$ as $k\rightarrow \infty$.

Despite the nonexistence of minimizers explained above, the energy functional ${\mathcal {H}}^{\lambda ,p}_{c_0}$ remains an important subject of study, since it is the critical points of ${\mathcal {H}}^{\lambda ,p}_{c_0}$ that are of interest.

### Diameter estimates

In this section, we will show that the Helfrich energy can be used to obtain bounds on the diameter.

#### Lemma 6.3

Suppose $V\in {\mathbb {V}}^{\textrm{o}}_2({\mathbb {R}}^3)$ and $H\in L^{2}_\textrm{loc}(\mu _V;{\mathbb {R}}^3)$ satisfy Hypothesis 2.2, ${{\,\textrm{spt}\,}}\mu _V$ is compact, and ${\mathcal {H}}_{c_0}(V)>0$. Then for all $x_0\in {{\,\textrm{spt}\,}}\mu _V$ we have

$$\begin{aligned} \frac{|2 \mu _V({\mathbb {R}}^3) -3c_0{{\,\mathrm{{\mathcal {V}}}\,}}(V,x_0)|}{2\sqrt{\mu _V({\mathbb {R}}^3){\mathcal {H}}_{c_0}(V)}}\le {{\,\textrm{diam}\,}}{{\,\textrm{spt}\,}}\mu _V. \end{aligned}$$

#### Proof

Using Hypothesis 2.2 for the vector field $X(x)=x-x_0$ (multiplied with a suitable cut-off function away from ${{\,\textrm{spt}\,}}\mu _V$), we have

$$\begin{aligned} \int \limits _{{\mathbb {R}}^3}2\mathop {}\!\textrm{d}\mu _V(x)-3c_0{{\,\mathrm{{\mathcal {V}}}\,}}(V,x_0)&= - \int \limits _{{\mathbb {G}}^{\textrm{o}}_2({\mathbb {R}}^3)}\langle H(x),x-x_0\rangle \mathop {}\!\textrm{d}V(x,\xi )\\&\quad +c_0 \int \limits _{{\mathbb {G}}^{\textrm{o}}_2({\mathbb {R}}^3)}\langle \star \xi ,x-x_0\rangle \mathop {}\!\textrm{d}V(x,\xi ). \end{aligned}$$

Thus, by the Cauchy–Schwarz inequality

$$\begin{aligned} \left|2\mu _V({\mathbb {R}}^3)-3c_0{{\,\mathrm{{\mathcal {V}}}\,}}(V,x_0)\right|&\le \int \limits _{{\mathbb {G}}^{\textrm{o}}_2({\mathbb {R}}^3)}|H(x)-c_0(\star \xi )||x-x_0|\mathop {}\!\textrm{d}V(x,\xi )\\&\le \sqrt{4{\mathcal {H}}_{c_0}(V)\mu _V({\mathbb {R}}^3)}{{\,\textrm{diam}\,}}{{\,\textrm{spt}\,}}\mu _V. \end{aligned}$$

$$\square $$

In the case $c_0=0$, this is just Simon’s lower diameter estimate, cf. [43, Lemma 1.1]. Note that here we did not use the Li–Yau inequality but merely the first variation formula, see (2.1) and (2.4).

#### Lemma 6.4

Suppose $V,H,E,\Theta$ satisfy Hypothesis 4.5, $\theta ^2(\mu _V,x)\ge 1$ for $\mu _V$-almost all *x*, ${{\,\textrm{spt}\,}}\mu _V$ is connected, and $c_0\le 0$. If ${\mathcal {H}}_{c_0}(V) < \infty$, $\mu _V({\mathbb {R}}^3)<\infty$, $\Theta \in L^1({\mathcal {L}}^3\llcorner E)$, and

6.2 $$\begin{aligned} \int \limits _E\frac{\Theta (x)}{|x-x_0|^2}\mathop {}\!\textrm{d}{\mathcal {L}}^3(x) < \infty \end{aligned}$$

for $\mu _V$-almost all $x_0$, then ${{\,\textrm{spt}\,}}\mu _V$ is compact and

6.3 $$\begin{aligned} {{\,\textrm{diam}\,}}{{\,\textrm{spt}\,}}\mu _V \le C \sqrt{{\mathcal {H}}_{c_0}(V)\Bigr (\mu _V({\mathbb {R}}^3) + \frac{2}{3}|c_0|{{\,\mathrm{{\mathcal {V}}}\,}}(V)\Bigl )} \end{aligned}$$

where $C = \frac{9}{2\pi }$ and ${{\,\mathrm{{\mathcal {V}}}\,}}(V) = \int _E\Theta \mathop {}\!\textrm{d}{\mathcal {L}}^3$ is the algebraic volume (see Proposition 4.8).

#### Remark 6.5

For $c_0 = 0$, we recover the diameter bound in terms of area and Willmore energy by Simon [43, Lemma 1.1]:

$$\begin{aligned} {{\,\textrm{diam}\,}}{{\,\textrm{spt}\,}}\mu _V \le C \sqrt{{\mathcal {W}}(V)\mu _V({\mathbb {R}}^3)}. \end{aligned}$$

This inequality holds true for all 2-varifolds in ${\mathbb {R}}^3$ with generalized perpendicular mean curvature, finite Willmore energy, and whose weight measure is finite and has connected support (see [39, Theorem 1.5]). Hence, by (2.5) we obtain

$$\begin{aligned} {{\,\textrm{diam}\,}}{{\,\textrm{spt}\,}}\mu _V \le C \sqrt{\mu _V({\mathbb {R}}^3)\Bigl (2{\mathcal {H}}_{c_0}(V) + \frac{1}{2}c_0^2\mu _V({\mathbb {R}}^3)\Bigr )} \end{aligned}$$

for all *V* satisfying Hypothesis 2.2 with ${\mathcal {W}}(V)<\infty$, $\mu _V({\mathbb {R}}^3)<\infty$, and such that ${{\,\textrm{spt}\,}}\mu _V$ is connected. In view of Remark 6.11, our diameter bound (6.3) will be particularly useful since it has the Helfrich functional rather than the weight measure as prefactor.

#### Proof of Lemma 6.4

We will follow the proof of [43, Lemma 1.1]. Suppose ${{\,\textrm{spt}\,}}\mu _V\ne \varnothing$ (otherwise the statement is trivial), let $x_0\in {{\,\textrm{spt}\,}}\mu _V$ and define the Radon measure

$$\begin{aligned} {\mathcal {H}}_{c_0}(V,B) :=\frac{1}{4}\int \limits _{B\times {\mathbb {G}}^{\textrm{o}}(3,2)}|H(x) - c_0(\star \xi )|^2\mathop {}\!\textrm{d}V(x,\xi ) \qquad \text {for all Borel sets }B\text { in }{\mathbb {R}}^3. \end{aligned}$$

Using the Cauchy–Schwarz inequality as in (4.13) and Young’s inequality, we estimate

$$\begin{aligned} \frac{1}{2\rho ^2}\int \limits _{B_\rho (x_0)\times \mathbb G^\textrm{o}(3,2)}\left| \langle x-x_0,H(x) - c_0(\star \xi )\rangle \right| \mathop {}\!\textrm{d}V(x,\xi ) \le \frac{\mu _V(B_\rho (x_0))}{2\rho ^2} + \frac{1}{2}{\mathcal {H}}_{c_0}(V,B_\rho (x_0)) \end{aligned}$$

for all $\rho >0$. Hence, since $\theta ^2(\mu _V,x_0)\ge 1$ by [39, Theorem 3.6] in combination with Remark 2.3, we can let $\sigma$ go to zero in Lemma 4.1 and use (4.13), (4.14) to infer

6.4 $$\begin{aligned} \pi&\le \frac{3}{4}{\mathcal {H}}_{c_0}(V,B_\rho (x_0))+\frac{3\mu _V(B_\rho (x_0))}{2\rho ^2} - \frac{c_0}{2}\int \limits _{B_\rho (x_0)\times \mathbb G^\textrm{o}(3,2)}\frac{\langle x-x_0,\star \xi \rangle }{|x-x_0|^2} \mathop {}\!\textrm{d}V(x,\xi ) \nonumber \\&\quad + \frac{c_0}{2\rho ^2}\int \limits _{B_\rho (x_0)\times \mathbb G^\textrm{o}(3,2)}\langle x-x_0,\star \xi \rangle \mathop {}\!\textrm{d}V(x,\xi ). \end{aligned}$$

Multiplying (4.24) with $\frac{c_0}{2}$ and using $c_0\le 0$, for any $0<\sigma <\rho$ we have

$$\begin{aligned}&- \frac{c_0}{2}\int \limits _{(B_\rho {\setminus } B_\sigma )(x_0)\times \mathbb G^\textrm{o}(3,2)}\frac{\langle x-x_0,\star \xi \rangle }{|x-x_0|^2} \mathop {}\!\textrm{d}V(x,\xi ) + \frac{c_0}{2\rho ^2}\int \limits _{B_\rho (x_0)\times \mathbb G^\textrm{o}(3,2)}\langle x-x_0,\star \xi \rangle \mathop {}\!\textrm{d}V(x,\xi ) \\&\quad = \frac{3|c_0|}{2\rho ^2}\int \limits _{E\cap B_\rho (x_0)} \Theta \mathop {}\!\textrm{d}{\mathcal {L}}^3 -\frac{|c_0|}{2} \int \limits _{E\cap B_\rho (x_0){\setminus } B_\sigma (x_0)}\frac{\Theta (x)}{|x-x_0|^2}\mathop {}\!\textrm{d}{\mathcal {L}}^3(x)\\&\qquad - \frac{3|c_0|}{2\sigma ^2}\int \limits _{E\cap B_\sigma (x_0)}\Theta \mathop {}\!\textrm{d}{\mathcal {L}}^3 - \frac{|c_0|}{2\sigma ^2}\int \limits _{B_\sigma (x_0)\times {\mathbb {G}}^{\textrm{o}}(3,2)}\langle x-x_0, \star \xi \rangle \mathop {}\!\textrm{d}V(x,\xi ). \end{aligned}$$

Sending $\sigma \rightarrow 0+$ and using (6.2), Lemma 3.2 and Remark 3.3(i), we find that

6.5 $$\begin{aligned}&-\frac{c_0}{2}\int \limits _{B_\rho (x_0)\times \mathbb G^\textrm{o}(3,2)}\frac{\langle x-x_0,\star \xi \rangle }{|x-x_0|^2} \mathop {}\!\textrm{d}V(x,\xi ) + \frac{c_0}{2\rho ^2}\int \limits _{B_\rho (x_0)\times \mathbb G^\textrm{o}(3,2)}\langle x-x_0,\star \xi \rangle \mathop {}\!\textrm{d}V(x,\xi ) \nonumber \\&= \frac{|c_0|}{2}\int \limits _{E\cap B_\rho (x_0)}\left( \frac{3}{\rho ^2} - \frac{1}{|x-x_0|^2}\right) \Theta (x) \mathop {}\!\textrm{d}{\mathcal {L}}^3(x) \le \frac{|c_0|}{\rho ^2}\int \limits _{E\cap B_\rho (x_0)}\Theta (x){\mathcal {L}}^3(x). \end{aligned}$$

Combining (6.4) and (6.5), we thus obtain

6.6 $$\begin{aligned} \pi \le \frac{3}{4}{\mathcal {H}}_{c_0}(V,B_\rho (x_0))+\frac{3}{2\rho ^2} \mu _V(B_\rho (x_0)) + \frac{|c_0|}{\rho ^2}\left( \Theta {\mathcal {L}}^3 \llcorner E\right) (B_\rho (x_0)). \end{aligned}$$

The right hand side of this inequality corresponds to the Radon measure

$$\begin{aligned} \mu _{c_0,V,E} := \frac{3}{4}{\mathcal {H}}_{c_0}(V,\cdot )+\frac{3}{2\rho ^2} \mu _V + \frac{|c_0|}{\rho ^2}\left( \Theta {\mathcal {L}}^3\llcorner E\right) . \end{aligned}$$

The set of $x_0\in {\mathbb {R}}^3$ that satisfy (6.2) is dense in ${{\,\textrm{spt}\,}}\mu _V$. Hence, given any $x_0\in {{\,\textrm{spt}\,}}\mu _V$ and any $\varepsilon >0$ we can always find $x_1\in {{\,\textrm{spt}\,}}\mu _V$ which satisfies (6.6) such that $B_\rho (x_1)\subset B_{\rho + \varepsilon }(x_0)$. Thus, letting $\varepsilon \rightarrow 0+$, we see that (6.6) remains valid for all $x_0\in {{\,\textrm{spt}\,}}\mu _V$. By Remark 3.3(i), we see $\mu _V(N) = 0$ whenever *N* is finite and consequently $\mu _{c_0,V,E}(N) = 0$ whenever *N* is finite. Let $d :={{\,\textrm{diam}\,}}{{\,\textrm{spt}\,}}\mu _V$ (possibly $d=\infty$), $0<\rho <d$, and *N* be a positive integer such that $2(N-1)\rho < d$. By the connectedness of ${{\,\textrm{spt}\,}}\mu _V$, we can choose points $x_0,\ldots ,x_{N-1}\in {{\,\textrm{spt}\,}}\mu _V$ such that $x_i \in \partial B_{2i\rho }(x_0)$ for $i=1,\ldots ,N-1$. The balls $B_\rho (x_0),\ldots ,B_\rho (x_{N-1})$ intersect in at most $N-1$ points. Applying the inequality (6.6) for each $x_i$ and summing over *i* yields

6.7 $$\begin{aligned} N\pi \le \mu _{c_0,V,E}({\mathbb {R}}^3). \end{aligned}$$

Since the right hand side is finite, it follows that the diameter *d* is finite. Hence, we can choose *N* such that $2(N-1)\rho <d\le 2N\rho$. Then (6.7) and Proposition 4.8 imply

6.8 $$\begin{aligned} d \le \frac{3}{2\pi }\left( \rho {\mathcal {H}}_{c_0}(V) + \frac{2}{\rho }\mu _V({\mathbb {R}}^3) + \frac{4|c_0|}{3\rho }{{\,\mathrm{{\mathcal {V}}}\,}}(V)\right) . \end{aligned}$$

Now, in view of Lemma 6.1, we may take

$$\begin{aligned} \rho = \sqrt{\frac{2\mu _V({\mathbb {R}}^3) + \frac{4}{3}|c_0|{{\,\mathrm{{\mathcal {V}}}\,}}(V)}{2{\mathcal {H}}_{c_0}(V)}} = \sqrt{\frac{\mu _V({\mathbb {R}}^3) + \frac{2}{3}|c_0|{{\,\mathrm{{\mathcal {V}}}\,}}(V)}{{\mathcal {H}}_{c_0}(V)}}. \end{aligned}$$

Then, by Lemma 6.3, $\rho < d$ and thus, (6.8) becomes

$$\begin{aligned} d \le \frac{9}{2\pi }\sqrt{{\mathcal {H}}_{c_0}(V)\Bigr (\mu _V({\mathbb {R}}^3) + \frac{2}{3}|c_0|{{\,\mathrm{{\mathcal {V}}}\,}}(V)\Bigl )} \end{aligned}$$

which concludes the proof. $\square$

### Regularity and embeddedness of Canham–Helfrich minimizers

We start with a survey of the variational setting in [33] (see also [22, 23, 31]). This includes the definition of *Lipschitz immersions*. Then we introduce the space ${\mathcal {Q}}_\Sigma$ of *Lipschitz quasi-embeddings* which consists of those Lipschitz immersions whose associated varifolds are varifolds with enclosed volume, cf. Hypothesis 4.5. We show that each injective Lipschitz immersion (in particular each smooth embedding) is a Lipschitz quasi-embedding (see Lemma 6.6). Moreover, we prove a weak closure Lemma 6.8 which leads to our main regularity Theorem 6.10.

Let $\Sigma$ be a closed oriented surface and let $g_0$ be a reference Riemannian metric on $\Sigma$. A map $f:\Sigma \rightarrow {\mathbb {R}}^3$ is called *weak branched immersion* if and only if

6.9 $$\begin{aligned} f \in W^{1, \infty }(\Sigma ; {\mathbb {R}}^3) \cap W^{2,2}(\Sigma ; {\mathbb {R}}^3), \end{aligned}$$

there exists a constant $1<C<\infty$ such that

6.10 $$\begin{aligned} C^{-1}|\mathrm df|_{g_0}\le |\mathrm df\wedge \mathrm df|_{g_0}\le C|\mathrm df|_{g_0} \end{aligned}$$

where in local coordinates

$$\begin{aligned} \mathrm df\wedge \mathrm df:=(\mathrm dx^1\wedge \mathrm dx^2)\partial _{x^1}f\wedge \partial _{x^2}f, \end{aligned}$$

there exist finitely many so called *branch points* $b_1, \ldots , b_N \in \Sigma$ such that the *conformal factor* satisfies

$$\begin{aligned} \log |\mathrm df|_{g_0} \in L^\infty _\textrm{loc}(\Sigma {\setminus } \{b_1, \ldots , b_N\}), \end{aligned}$$

and the Gauss map *n* defined as in (2.6) satisfies

6.11 $$\begin{aligned} n \in W^{1,2}(\Sigma ; {\mathbb {R}}^3). \end{aligned}$$

If in addition

6.12 $$\begin{aligned} |\partial _{x^1} f| = |\partial _{x^2} f| \quad \text {and}\quad \langle \partial _{x^1} f, \partial _{x^2} f\rangle = 0 \end{aligned}$$

for all conformal charts *x* of $(\Sigma ,g_0)$, then *f* is called *conformal*. A chart $x = (x^1,x^2)$ that satisfies (6.12) is referred to as *isothermal coordinates*. Notice that (6.12) implies (6.10) and, since $\Sigma$ is closed, the conditions (6.9)–(6.11) do not depend on the choice of the Riemannian metric $g_0$. The space of weak branched immersions is denoted by ${\mathcal {F}}_{\Sigma }$. The subspace ${\mathcal {E}}_\Sigma$ of *Lipschitz immersions* is defined to consist of all $f\in {\mathcal {F}}_{\Sigma }$ such that there exists a constant $0<C<\infty$ with

6.13 $$\begin{aligned} |\mathrm df\wedge \mathrm df|_{g_0} \ge C. \end{aligned}$$

Notice that (6.9) and (6.13) imply $\log |\mathrm df|_{g_0}\in L^\infty (\Sigma )$.

Let $f\in {\mathcal {F}}_\Sigma$. Analogously to Example 2.4, we infer a (possibly degenerated) $L^\infty$-metric $g:=f^*\langle \cdot ,\cdot \rangle$, the induced Radon measure $\mu$ over $\Sigma$, the oriented varifold $V:=(f,\star n)_\#\mu$, the classical mean curvature $H_f$ of *f* (in the Sobolev sense), and the induced generalized mean curvature *H*. If *f* is conformal, we have by [23, Theorem 3.1] that

$$\begin{aligned} {\mathcal {H}}^0(f^{-1}\{x\}) = \theta ^2(\mu _V,x) \qquad \text {for all }x\in {\mathbb {R}}^3. \end{aligned}$$

In view of [31, Equation (2.11)] there holds

$$\begin{aligned} \delta V(X) = - \int \limits _{{\mathbb {R}}^3}\langle X,H\rangle \mathop {}\!\textrm{d}\mu _V \end{aligned}$$

for all $X\in C^1_{c}({\mathbb {R}}^3;{\mathbb {R}}^3)$. Moreover, by the definition of *H* and (6.11) we have that

$$\begin{aligned} \int \limits _{{\mathbb {R}}^3}|H|^2\mathop {}\!\textrm{d}\mu _V \le \int \limits _{\Sigma } |H_f|^2\mathop {}\!\textrm{d}\mu < \infty . \end{aligned}$$

In particular, $H \in L^2(\mu _V;{\mathbb {R}}^3)$ and *V*, *H* satisfy Hypothesis 2.2. Now, we can combine [17, Section 6.1, Theorem 4] and [40, Theorem 4.1] to infer

$$\begin{aligned} H(f(p)) = H_f(p) \qquad \text {for }\mu \text {-almost all }p\in \Sigma . \end{aligned}$$

As in (2.9), it follows that for all $c_0 \in {\mathbb {R}}$ we have

6.14 $$\begin{aligned} {\mathcal {H}}_{c_0}(V) = \frac{1}{4}\int \limits _{{\mathbb {R}}^3}|H(x) - c_0(\star \xi )|^2\mathop {}\!\textrm{d}V(x,\xi ) = \frac{1}{4}\int \limits _{\Sigma }|H_f - c_0n|^2\mathop {}\!\textrm{d}\mu = {\mathcal {H}}_{c_0}(f).\quad \end{aligned}$$

The space ${\mathcal {Q}}_\Sigma$ is defined to consist of all $f\in {\mathcal {E}}_\Sigma$ such that there exists an ${\mathcal {L}}^3$-measurable set *E* with

6.15 $$\begin{aligned} {{\,\textrm{diam}\,}}{{\,\textrm{spt}\,}}({\mathcal {L}}^3\llcorner E) \le {{\,\textrm{diam}\,}}f[\Sigma ] \end{aligned}$$

and

6.16 $$\begin{aligned} \int \limits _E {{\,\textrm{div}\,}}X\mathop {}\!\textrm{d}{\mathcal {L}}^3 =- \int \limits _{\Sigma }\langle X\circ f, n\rangle \mathop {}\!\textrm{d}\mu \end{aligned}$$

for any Lipschitz map $X:{\mathbb {R}}^3\rightarrow {\mathbb {R}}^3$ with compact support, i.e. the triple *E*, *V*, *H* satisfies Hypothesis 4.5 for $\Theta \equiv 1$. The divergence theorem for sets of finite perimeter (4.16), Eq. (6.16), and the area formula (see [18, 3.2.22(3)]) imply

6.17 $$\begin{aligned} n_E(x) = \sum _{p\in f^{-1}\{x\}} n(p) \qquad \text {for }{\mathcal {H}}^2\text {-almost all }x\in {\mathbb {R}}^3. \end{aligned}$$

Notice that $x\notin {{\,\textrm{spt}\,}}({\mathcal {H}}^2\llcorner \partial _*E)$ does not imply $f^{-1}\{x\} = \varnothing$. In particular, in view of Fig. 2e, f, the two oriented varifolds associated with $\partial _*E$ and *f* do not necessarily coincide. Hence, by Proposition 3.4, Proposition 4.8, Lemma 4.9 and Remark 4.10 there holds

6.18 $$\begin{aligned} {{\,\mathrm{{\mathcal {V}}}\,}}(V,x_0) = {{\,\mathrm{{\mathcal {V}}}\,}}(f) = {\mathcal {L}}^3(E),\qquad {{\,\mathrm{{\mathcal {V}}}\,}}_c(V,x_0) = {{\,\mathrm{{\mathcal {V}}}\,}}_c(f,x_0) = \int \limits _E\frac{1}{|x-x_0|^2}\mathop {}\!\textrm{d}{\mathcal {L}}^3(x)\qquad \end{aligned}$$

for all $x_0\in {\mathbb {R}}^3$. If ${\mathcal {H}}^0(f^{-1}\{x\}) \le 1$ for ${\mathcal {H}}^2$-almost all $x\in {\mathbb {R}}^3$ then (6.17) implies $n_E \circ f = n$, $\partial _*E = f[\Sigma ]$ up to a set of ${\mathcal {H}}^2$-measure zero, the two oriented varifolds associated with $\partial _*E$ and *f* coincide, and since ${{\,\textrm{spt}\,}}({\mathcal {H}}^2\llcorner \partial _*E) \subset {{\,\textrm{spt}\,}}({\mathcal {L}}^3\llcorner E)$, equality holds in (6.15). Inspired by the terminology in the smooth case of [4], we refer to ${\mathcal {Q}}_\Sigma$ as the space of *Lipschitz quasi-embeddings*. By definition, there holds

$$\begin{aligned} {\mathcal {Q}}_\Sigma \subset {\mathcal {E}}_\Sigma \subset {\mathcal {F}}_{\Sigma }. \end{aligned}$$

#### Lemma 6.6

Let $\Sigma$ be a closed oriented surface and $f\in {\mathcal {E}}_\Sigma$ be injective. Then, possibly after changing the orientation of $\Sigma$, there exists a connected open bounded set $U\subset {\mathbb {R}}^3$ of finite perimeter such that $\partial _*U = f[\Sigma ]$ up a to a set of ${\mathcal {H}}^2$-measure zero, and

6.19 $$\begin{aligned} \int \limits _U {{\,\textrm{div}\,}}X\mathop {}\!\textrm{d}{\mathcal {L}}^3 =- \int \limits _{\Sigma }\langle X\circ f, n\rangle \mathop {}\!\textrm{d}\mu \end{aligned}$$

for any Lipschitz map $X:{\mathbb {R}}^3\rightarrow {\mathbb {R}}^3$. In particular, $f\in {\mathcal {Q}}_\Sigma$ and ${\mathcal {Q}}_\Sigma$ contains all smooth embeddings $f:\Sigma \rightarrow {\mathbb {R}}^3$ (up to orientation). However, not all $f\in {\mathcal {Q}}_\Sigma$ are injective.

#### Remark 6.7

Notice that changing the orientation on $\Sigma$ is equivalent to changing the sign of the (nonzero) algebraic volume. Hence, if additionally ${{\,\mathrm{{\mathcal {V}}}\,}}(f)>0$, no change of orientation is necessary in Lemma 6.6.

#### Proof of Lemma 6.6

We may assume that $(\Sigma ,g_0)\subset {\mathbb {R}}^3$ is embedded and $g_0$ is the metric induced by the inclusion map. Since *f* is injective, we can apply the Jordan–Brouwer separation theorem [9] to obtain a connected open bounded set $U\subset {\mathbb {R}}^3$ such that $\partial U = f[\Sigma ]$ and ${\mathbb {R}}^3{\setminus } {\bar{U}}$ is connected. Since $\partial _*U\subset \partial U = f[\Sigma ]$ and ${\mathcal {H}}^2(f[\Sigma ])<\infty$, Federer’s criterion implies that *U* is a set of finite perimeter. Moreover, for $p\in \Sigma$, one can show that if *f* is differentiable at *p*, then $f(p)\in \partial _*U$. Hence, by Rademacher’s theorem, the sets $\partial _*U$ and $f[\Sigma ]$ are ${\mathcal {H}}^2$-almost equal. We still need to show that

$$\begin{aligned} \int \limits _{\partial _*U}\langle X,n_U\rangle \mathop {}\!\textrm{d}{\mathcal {H}}^2 = \int \limits _{\Sigma }\langle X\circ f,n\rangle \mathop {}\!\textrm{d}\mu \end{aligned}$$

for all Lipschitz maps $X:{\mathbb {R}}^3\rightarrow {\mathbb {R}}^3$, where $n_U$ is the measure theoretic inner unit normal of *U* (see Sect. 4.3), and *n* is the Gauss map of *f*, cf. (2.6). Let $\nu$ be the unit normal induced by the orientation of $\Sigma \subset {\mathbb {R}}^3$. We define the 2-current *T* on ${\mathbb {R}}^3$ by

$$\begin{aligned} T(\omega ) :=- \int \limits _\Sigma \omega _p(\star \nu (p))\mathop {}\!\textrm{d}{\mathcal {H}}^2(p) \end{aligned}$$

for all differential forms $\omega$ of degree 2 on ${\mathbb {R}}^3$. Since $\Sigma$ is closed, we have

6.20 $$\begin{aligned} \partial T = 0 \end{aligned}$$

(see for instance [18, 4.1.31(1)]). Given any positive chart *x* of $\Sigma$, there holds

$$\begin{aligned} \nu = \frac{\partial _{x^1}\times \partial _{x^2}}{|\partial _{x^1}\times \partial _{x^2}|},\qquad ({\textstyle \bigwedge }_2\mathrm df)(\star \nu ) = \frac{|\partial _{x^1}f\wedge \partial _{x^2}f|}{|\partial _{x^1}\times \partial _{x^2}|}(\star n) \end{aligned}$$

where for ${\mathcal {H}}^2$-almost all $p\in \Sigma$, the linear map ${\textstyle \bigwedge }_2\mathrm df_p:{\textstyle \bigwedge }_2 T_p\Sigma \rightarrow {\textstyle \bigwedge }_2 \mathrm df_p[T_p\Sigma ]$ is defined as in [18, 1.3.1]. Recalling that in any local chart *x*, the area elements of the immersion *f* and the inclusion $\Sigma \subset {\mathbb {R}}^3$ are given by $|\partial _{x^1}f\wedge \partial _{x^2}f|$ and $|\partial _{x^1}\times \partial _{x^2}|$, respectively, we have by [18, 4.1.30] that

$$\begin{aligned} R(\omega ):=(f_\#T)(\omega ) = -\int \limits _{\Sigma }\omega _{f(p)}(\star n(p))\mathop {}\!\textrm{d}\mu (p) \end{aligned}$$

for all differential forms $\omega$ of degree 2 on ${\mathbb {R}}^3$. Thus, by [18, 4.1.14] and (6.20)

$$\begin{aligned} \partial R = \partial (f_\#T) = f_\#(\partial T) = 0. \end{aligned}$$

Therefore, we can combine [18, 4.5.17] and [18, 4.5.6] to deduce the existence of sets of finite perimeter $E_j\subset E_{j-1}\subset {\mathbb {R}}^3$, $j\in {\mathbb {Z}}$ such that

6.21 $$\begin{aligned} R = \sum _{j\in {\mathbb {Z}}} R_j,\qquad \mu _V = \sum _{j\in {\mathbb {Z}}}({\mathcal {H}}^2\llcorner \partial _*E_j) \end{aligned}$$

where

$$\begin{aligned} R_j(\omega ):=- \int \limits _{\partial _*E_j}\omega _x(\star n_{E_j}(x))\mathop {}\!\textrm{d}{\mathcal {H}}^2(x) \end{aligned}$$

are the currents induced by $\partial _*E_j$. Since *U* is open and connected, we see from [3, Proposition 2] that *U* is indecomposable. Given any set of finite perimeter $E\subset {\mathbb {R}}^3$ with $\partial _*E \subset \partial _*U$ up to a set of ${\mathcal {H}}^2$-measure zero, we see that $\partial _*(U\cap E) \subset \partial _*U$ up to a set of ${\mathcal {H}}^2$-measure zero and thus, by [3, Proposition 4], either ${\mathcal {L}}^3(U \cap E) = 0$ or ${\mathcal {L}}^3(U{\setminus } E)=0$. The same holds true for *U* replaced by ${\mathbb {R}}^3{\setminus } {\bar{U}}$. By (6.21) we have for all $j\in {\mathbb {Z}}$ that $\partial _*E_j\subset {{\,\textrm{spt}\,}}\mu _V = \partial _*U$ up to a set of ${\mathcal {H}}^2$-measure zero and therefore either $E_j={\mathbb {R}}^3$ or $E_j = U$ or $E_j = {\mathbb {R}}^3{\setminus } {\bar{U}}$ or $E_j = \varnothing$ up to a set of ${\mathcal {L}}^3$-measure zero. Since *f* is injective, we have that $\theta ^2(\mu _V,\cdot )\le 1$. We thus deduce the existence of $j_0\in {\mathbb {Z}}$ such that (up to a set of ${\mathcal {L}}^3$-measure zero and possibly after changing the orientation on $\Sigma$)

$$\begin{aligned} E_j = {\left\{ \begin{array}{ll} {\mathbb {R}}^3 &{}\text {for }j<j_0,\\ U &{}\text {for }j=j_0, \\ \varnothing &{}\text {for }j>j_0. \end{array}\right. } \end{aligned}$$

In particular, $R=R_{j_0}$ and (6.19) follows. To see that not all $f\in {\mathcal {Q}}_\Sigma$ are injective, one may consider surfaces like in Fig. 2b, d, e. $\square$

In the following, we abbreviate ${\mathcal {F}}:={\mathcal {F}}_{{\mathbb {S}}^2}$, ${\mathcal {E}}:={\mathcal {E}}_{{\mathbb {S}}^2}$, and ${\mathcal {Q}}:=\mathcal Q_{{\mathbb {S}}^2}$.

#### Lemma 6.8

Suppose $f_k$ is a sequence in ${\mathcal {Q}}$, $0\in f_k[{\mathbb {S}}^2]$ for all $k\in {\mathbb {N}}$, $c_0\in {\mathbb {R}}$,

6.22 $$\begin{aligned} A_0:=\sup _{k\in {\mathbb {N}}} {\mathcal {A}}(f_k)<\infty , \qquad \inf _{k\in {\mathbb {N}}}{{\,\textrm{diam}\,}}f_k[{\mathbb {S}}^2] >0, \end{aligned}$$

and

6.23 $$\begin{aligned} {\left\{ \begin{array}{ll} \liminf _{k\rightarrow \infty }\bigl ({\mathcal {H}}_{c_0}(f_k) +2c_0\inf _{x\in f_k[{\mathbb {S}}^2]}{{\,\mathrm{{\mathcal {V}}}\,}}_c(f_k,x)\bigr )< 8\pi &{} \text {if }c_0<0,\\ \liminf _{k\rightarrow \infty }\bigl ({\mathcal {H}}_{c_0}(f_k) +2c_0\sup _{x\in f_k[{\mathbb {S}}^2]}{{\,\mathrm{{\mathcal {V}}}\,}}_c(f_k,x)\bigr ) < 8\pi &{} \text {if }c_0\ge 0. \end{array}\right. } \end{aligned}$$

Then, after passing to a subsequence, there exists $f\in {\mathcal {Q}}$ injective such that

6.24 $$\begin{aligned} V_k\rightarrow V\qquad \text {in }{\mathbb {V}}_2^\textrm{o}({\mathbb {R}}^3)\text { as }k\rightarrow \infty , \end{aligned}$$

where $V_k,V$ are the oriented 2-varifolds in ${\mathbb {R}}^3$ associated with $f_k,f$ (cf. Example 2.4) and

6.25 $$\begin{aligned} {\mathcal {H}}_{c_0}(f)\le \liminf _{k\rightarrow \infty }{\mathcal {H}}_{c_0}(f_k). \end{aligned}$$

#### Proof

Let $g_0$ be the standard metric on ${\mathbb {S}}^2$. By [33, Theorem 1.4], after reparametrization, we may assume that all $f_k$ are conformal. After passing to a subsequence, we may further assume that for all $k\in {\mathbb {N}}$,

$$\begin{aligned} {\left\{ \begin{array}{ll} {\mathcal {H}}_{c_0}(f_k) +2c_0\inf _{x\in f_k[{\mathbb {S}}^2]}{{\,\mathrm{{\mathcal {V}}}\,}}_c(f_k,x)< 8\pi &{} \text {if }c_0<0,\\ {\mathcal {H}}_{c_0}(f_k) +2c_0\sup _{x\in f_k[{\mathbb {S}}^2]}{{\,\mathrm{{\mathcal {V}}}\,}}_c(f_k,x) < 8\pi &{} \text {if }c_0\ge 0. \end{array}\right. } \end{aligned}$$

Let $E_k$ be the sequence of sets of finite perimeter corresponding to $f_k$ according to (6.16). Using (6.18), for all $x_0\in {\mathbb {R}}^3$ and $k\in {\mathbb {N}}$ there holds

$$\begin{aligned} {{\,\mathrm{{\mathcal {V}}}\,}}_c(f_k,x_0) \le \int \limits _{B_1(x_0)}\frac{1}{|x-x_0|^2}\mathop {}\!\textrm{d}{\mathcal {L}}^3(x) + {\mathcal {L}}^3(E_k) = 4\pi + {{\,\mathrm{{\mathcal {V}}}\,}}(f_k) \end{aligned}$$

and thus, by (6.15) we can apply the isoperimetric inequality for sets of finite perimeter (see [18, Corollary 4.5.3]) to deduce from the uniform area bound (6.22) that

6.26 $$\begin{aligned} V_0:=\sup _{k\in {\mathbb {N}}}{{\,\mathrm{{\mathcal {V}}}\,}}(f_k)<\infty ,\qquad \sup _{k\in {\mathbb {N}}}\sup _{x\in {\mathbb {R}}^3}{{\,\mathrm{{\mathcal {V}}}\,}}_c(f_k,x)<C(V_0)<\infty . \end{aligned}$$

Hence, by [31, Equation (2.8)] and (2.5), there holds

6.27 $$\begin{aligned} \int \limits _{{\mathbb {S}}^2}1 + |\mathrm dn_{f_k}|_{g_0}^2\mathop {}\!\textrm{d}\mu _{f_k}&\le A_0 + 4{\mathcal {W}}(f_k) \le A_0 + 8{\mathcal {H}}_{c_0}(f_k) + 2c_0^2A_0 \nonumber \\&\le A_0 + 8\bigl (8\pi + 2|c_0|C(V_0) + c_0^2A_0\bigr ) \end{aligned}$$

for all $k\in {\mathbb {N}}$. Therefore, we can apply [31, Theorem 3.3] (see also Theorem 1.5 and Lemma 4.1 in [30]) to infer that after passing to a subsequence, there exist a positive integer *N* and sequences $\phi _k^1,\ldots ,\phi _k^N$ of positive conformal $C^\infty$-diffeomorphisms of ${\mathbb {S}}^2$ such that for each $i\in \{1,\ldots ,N\}$, there exist $f^i\in {\mathcal {F}}_{{\mathbb {S}}^2}$ conformal, $N^i\in {\mathbb {N}}$, and finitely many points $b^{i,1},\ldots ,b^{i,N^i}\in {\mathbb {S}}^2$ with

6.28 $$\begin{aligned}{} & {} f^i_k:=f_k\circ \phi ^i_k\rightharpoonup f^i \qquad \text {weakly in }W^{2,2}_\textrm{loc}({\mathbb {S}}^2{\setminus }\{b^{i,1},\ldots ,b^{i,N^i}\};{\mathbb {R}}^3)\text { as }k\rightarrow \infty ,\qquad \end{aligned}$$

6.29 $$\begin{aligned}{} & {} \sup _{k\in {\mathbb {N}}}\Vert \log |\mathrm df_k^i|_{g_0}\Vert _{L^\infty _{\textrm{loc}}({\mathbb {S}}^2{\setminus }\{b^{i,1},\ldots ,b^{i,N^i}\})}<\infty . \end{aligned}$$

Moreover, there exist a sequence $\psi _k$ of $C^\infty$-diffeomorphisms of ${\mathbb {S}}^2$ and $f\in W^{1,\infty }({\mathbb {S}}^2;{\mathbb {R}}^3)$ such that

6.30 $$\begin{aligned} f_k\circ \psi _k \rightarrow f \quad \text {in }C^0({\mathbb {S}}^2;{\mathbb {R}}^3)\text { as }k\rightarrow \infty ,\qquad f[{\mathbb {S}}^2] = \bigcup _{i=1}^Nf^i[{\mathbb {S}}^2]. \end{aligned}$$

Furthermore, there holds

6.31 $$\begin{aligned} \sum _{i=1}^N{\mathcal {W}}(f^i) \le \liminf _{k\rightarrow \infty }\mathcal {W}(f_k),\qquad \sum _{i=1}^N{\mathcal {H}}_{c_0}(f^i)\le \liminf _{k\rightarrow \infty }{\mathcal {H}}_{c_0}(f_k). \end{aligned}$$

Denote with $V^i$ the varifolds associated to $f^i$ and set $V:=\sum _{i=1}^NV^i$. In order to show (6.24), let $\varphi :{\mathbb {R}}^3\times {\mathbb {G}}^\textrm{o}(3,2)\rightarrow {\mathbb {R}}$ be any continuous function with compact support. Fix an integer $i\in \{1,\ldots ,N\}$, choose a conformal chart $x:{\mathbb {S}}^2{\setminus }\{b^{i,1},\ldots ,b^{i,N^i}\}\rightarrow {\mathbb {R}}^2$, and let $K\subset {\mathbb {S}}^2{\setminus }\{b^{i,1},\ldots ,b^{i,N^i}\}$ be a compact set. Denote by

$$\begin{aligned} \lambda _k^i :=\log |\partial _{x^1}f_k^i|,\qquad \lambda ^i :=\log |\partial _{x^1}f^i| \end{aligned}$$

the conformal factors and recall that the area elements of $f_k^i$ and $f^i$ are given by $e^{2\lambda _k^i}$ and $e^{2\lambda ^i}$. Let $n_k^i$, $n_k$, and $n^i$ be the Gauss maps of $f_k^i$, $f_k$, and $f^i$. Following the proof of [31, Lemma 3.1], we infer that by the weak convergence (6.28), the Rellich–Kondrachov compactness theorem, and the uniform bounds on the conformal factors (6.29), after passing to a subsequence,

6.32 $$\begin{aligned}&e^{2\lambda _k^i}\circ x^{-1}\rightarrow e^{2\lambda ^i}\circ x^{-1}{} & {} \text {in }L^p(x[K])\text { as }k\rightarrow \infty \text { for all }1\le p<\infty , \nonumber \\&f_k^i\circ x^{-1}\rightarrow f^i\circ x^{-1}{} & {} \text {pointwise almost everywhere on }x[K]\text { as }k\rightarrow \infty ,\nonumber \\&n_k^i\circ x^{-1}\rightarrow n^i\circ x^{-1}{} & {} \text {pointwise almost everywhere on }x[K]\text { as }k\rightarrow \infty . \end{aligned}$$

Hence, since $\varphi$ is continuous, and also the Hodge star operator $\star$ is continuous,

$$\begin{aligned} \varphi (f_k^i,\star n_k^i)\circ x^{-1} \rightarrow \varphi (f^i,\star n^i)\circ x^{-1} \qquad \text {pointwise almost everywhere on }x[K] \end{aligned}$$

as $k\rightarrow \infty$. Thus, since $\varphi$ is bounded, the dominated convergence theorem and (6.32) imply

$$\begin{aligned} \left( \varphi (f_k^i,\star n_k^i) e^{2\lambda _k^i}\right) \circ x^{-1}\rightarrow \left( \varphi (f^i,\star n^i) e^{2\lambda ^i}\right) \circ x^{-1} \qquad \text {in }L^p(x[K])\text { as }k\rightarrow \infty \end{aligned}$$

for any $1\le p < \infty$. Therefore, inductively passing to a subsequence, we can achieve that for all $k_0\in {\mathbb {N}}$ and all $k_0\le k\in {\mathbb {N}}$, there holds

6.33 $$\begin{aligned} \int \limits _{x\bigl [{\mathbb {S}}^2{\setminus }\bigcup _{j=1}^{N^i}B_{\frac{1}{k_0}}(b^{i,j})\bigr ]}\left| \varphi (f_k^i,\star n_k^i) e^{2\lambda _k^i} - \varphi (f^i,\star n^i) e^{2\lambda ^i} \right| \circ x^{-1}\mathop {}\!\textrm{d}{\mathcal {L}}^2 \le \frac{1}{k_0}. \end{aligned}$$

Successively passing to a subsequence, we infer that (6.33) holds true simultaneously for all $i\in \{1,\ldots ,N\}$. (Notice however that the chart *x* actually depends on *i*.) Moreover, since $\varphi$ is bounded and by the fact that finite sets have $\mu _{f_i}$-measure zero by Remark 3.3(i), there holds

$$\begin{aligned} \lim _{k_0\rightarrow \infty }\int \limits _{\bigcup _{j=1}^{N^i}B_{\frac{1}{k_0}}(b^{i,j})}\varphi (f^i,\star n^i)\mathop {}\!\textrm{d}\mu _{f^i} = 0, \end{aligned}$$

for all $i\in \{1, \dots ,N\}$. Writing $s_k:=1/k$, by (2.7) it follows

6.34 $$\begin{aligned} \int \limits _{{\mathbb {G}}_2^\textrm{o}({\mathbb {R}}^3)}\varphi \mathop {}\!\textrm{d}V^i = \int \limits _{{\mathbb {S}}^2}\varphi (f^i,\star n^i)\mathop {}\!\textrm{d}\mu _{f^i} = \lim _{k\rightarrow \infty }\int \limits _{{\mathbb {S}}^2{\setminus }\bigcup _{j=1}^{N^i}B_{s_k}(b^{i,j})}\varphi (f_k^i,\star n_k^i)\mathop {}\!\textrm{d}\mu _{f_k^i}. \end{aligned}$$

By the proof of [31, Theorem 3.3] (see also the proof of [30, Theorem 1.5]), there exist Borel sets $S^{i,j}_k\subset {\mathbb {S}}^2$ such that (see Equations (3.19) and (3.20) in [31])

6.35 $$\begin{aligned} \lim _{k\rightarrow \infty }\int \limits _{S^{i,j}_k}1\mathop {}\!\textrm{d}\mu _{f_k^i} = 0 \qquad \text {for all }i\in \{1,\ldots ,N\}\text { and }j\in \{1,\ldots ,N^{i}\} \end{aligned}$$

and

6.36 $$\begin{aligned} \int \limits _{{\mathbb {G}}_2^\textrm{o}({\mathbb {R}}^3)}\varphi \mathop {}\!\textrm{d}V_k = \int \limits _{{\mathbb {S}}^2}\varphi (f_k,\star n_k)\mathop {}\!\textrm{d}\mu _{f_k}&= \sum _{i=1}^N \int \limits _{{\mathbb {S}}^2{\setminus }\bigcup _{j=1}^{N^i}B_{s_k}(b^{i,j})}\varphi (f_k^i,\star n_k^i)\mathop {}\!\textrm{d}\mu _{f_k^i} \nonumber \\&\quad + \sum _{i=1}^N\sum _{j=1}^{N^i-1} \int \limits _{S_k^{i,j}}\varphi (f_k^i,\star n_k^i)\mathop {}\!\textrm{d}\mu _{f_k^i}. \end{aligned}$$

By (6.35) and the boundedness of $\varphi$, there holds

$$\begin{aligned} \left| \int \limits _{S_k^{i,j}}\varphi (f_k^i,\star n_k^i)\mathop {}\!\textrm{d}\mu _{f_k^i}\right| \le \Vert \varphi \Vert _{C^0(\mathbb G_2^\textrm{o}({\mathbb {R}}^3))}\int \limits _{S_k^{i,j}}1\mathop {}\!\textrm{d}\mu _{f_k^i}\rightarrow 0\qquad \text {as }k\rightarrow \infty . \end{aligned}$$

Thus, (6.36) and (6.34) imply

$$\begin{aligned} \lim _{k\rightarrow \infty }\int \limits _{{\mathbb {G}}_2^\textrm{o}({\mathbb {R}}^3)}\varphi \mathop {}\!\textrm{d}V_k = \sum _{i=1}^N\int \limits _{{\mathbb {G}}_2^\textrm{o}({\mathbb {R}}^3)}\varphi \mathop {}\!\textrm{d}V^i = \int \limits _{{\mathbb {G}}_2^\textrm{o}({\mathbb {R}}^3)}\varphi \mathop {}\!\textrm{d}V \end{aligned}$$

which proves (6.24).

By (6.27) there holds

$$\begin{aligned} D_0:=\sup _{k\in {\mathbb {N}}}{\mathcal {W}}(f_k) < \infty . \end{aligned}$$

Thus, by Lemma 3.7, there exists a constant $C(A_0,D_0)$ depending only on the energy bound $D_0$ and the area bound $A_0$ in (6.22) such that

6.37 $$\begin{aligned} |{{\,\mathrm{{\mathcal {V}}}\,}}_c(f_k,x) - {{\,\mathrm{{\mathcal {V}}}\,}}_c(f_k,y)|\le C(A_0,D_0)|x-y|^{1/2}\quad \text {for all }k\in {\mathbb {N}}\text { and all }x,y\in {\mathbb {R}}^3.\qquad \end{aligned}$$

Hence, by the varifold convergence (6.24), we can apply Lemma 3.6 and (6.30) to deduce first

6.38 $$\begin{aligned} \lim _{k\rightarrow \infty } {{\,\mathrm{{\mathcal {V}}}\,}}_c(V_k,f_k(p)) = {{\,\mathrm{{\mathcal {V}}}\,}}_c(V,f(p)) \qquad \text {for all }p\in {\mathbb {S}}^2 \end{aligned}$$

and secondly, by (6.23), the lower semi-continuity (6.31), and (6.14)

$$\begin{aligned} {\mathcal {H}}_{c_0}(V) + 2 c_0{{\,\mathrm{{\mathcal {V}}}\,}}_c(V,x_0) < 8\pi \qquad \text {for all }x_0\in {{\,\textrm{spt}\,}}\mu _V. \end{aligned}$$

Therefore, we can apply the Li–Yau inequality for general varifolds Corollary 4.4 to infer $\theta ^2(\mu _V,\cdot )< 2$. Now, it follows from (6.30) that $f=f^1\in {\mathcal {F}}$ and *f* is injective. In particular, (6.25) follows from (6.31). Moreover, by [23, Theorem 3.1], *f* has no branch points. That is $\log |\mathrm d f|_{g_0} \in L^\infty ({\mathbb {S}}^2)$ and thus $f\in {\mathcal {E}}$. It remains to show that $f\in {\mathcal {Q}}$. Recalling that $\{ x \in {\mathbb {R}}^3 \mid n_{E_k}(x)\ne 0\} = \partial _* E_k$ up to a set of ${\mathcal {H}}^2$-measure zero, we see from (6.17) that $\partial _* E_k\subset f_k[{\mathbb {S}}^2]$ up to a set of ${\mathcal {H}}^2$-measure zero, and thus ${\mathcal {H}}^{2}(\partial _* E_k) \le {\mathcal {A}}(f_k)$. Hence, the uniform area bound (6.22) and the uniform volume bound (6.26) imply that the sequence $\chi _{E_k}$ is bounded in $BV({\mathbb {R}}^3)$. Therefore, by compactness (see [17, Section 5.2, Theorem 4]), there exists an ${\mathcal {L}}^3$-measurable set *E* of of finite perimeter such that, after passing to a subsequence, $\chi _{E_k}\rightarrow \chi _E$ in $L^1({\mathbb {R}}^3)$ and pointwise almost everywhere as $k\rightarrow \infty$. In particular, the left hand side in (6.16) converges as $k\rightarrow \infty$. Moreover, the right hand side of (6.16) converges by (2.7) as a consequence of the varifold convergence (6.24). Noting that ${\mathcal {L}}^3\llcorner E_k \rightarrow {\mathcal {L}}^3\llcorner E$ as Radon measures for $k\rightarrow \infty$, we see that by (6.15) and the $C^0$-convergence (6.30)

$$\begin{aligned} {{\,\textrm{diam}\,}}{{\,\textrm{spt}\,}}({\mathcal {L}}^3\llcorner E) \le \liminf _{k\rightarrow \infty } {{\,\textrm{diam}\,}}f_k[{\mathbb {S}}^2] \le {{\,\textrm{diam}\,}}f[{\mathbb {S}}^2]. \end{aligned}$$

Thus, $f\in {\mathcal {Q}}$ and the proof is concluded. $\square$

#### Remark 6.9

The minimizer in [31, Theorem 1.7] has positive algebraic volume $V_0$. However, in view of Example 3.8 this is in general not enough to deduce that also the concentrated volume is nonnegative. Thus, we could not apply the Li–Yau inequality Corollary 4.4 directly to the minimizer in [31, Theorem 1.7].

#### Theorem 6.10

Suppose $c_0\in {\mathbb {R}}$, the numbers $A_0,V_0>0$ satisfy the isoperimetric inequality $36\pi V_0^2\le A_0^3$, and there exists a minimizing sequence $f_k$ of

6.39 $$\begin{aligned} {\bar{\eta }}(c_0,A_0,V_0):=\inf \{{\mathcal {H}}_{c_0}(f)\mid f\in \mathcal Q,\,{\mathcal {A}}(f) = A_0,\,{{\,\mathrm{{\mathcal {V}}}\,}}(f)=V_0\} \end{aligned}$$

such that

6.40 $$\begin{aligned} {\left\{ \begin{array}{ll} \liminf _{k\rightarrow \infty }\bigl ({\mathcal {H}}_{c_0}(f_k) +2c_0\inf _{x\in f_k[{\mathbb {S}}^2]}{{\,\mathrm{{\mathcal {V}}}\,}}_c(f_k,x)\bigr )< 8\pi &{} \text {if }c_0<0,\\ \liminf _{k\rightarrow \infty }\bigl ({\mathcal {H}}_{c_0}(f_k) +2c_0\sup _{x\in f_k[{\mathbb {S}}^2]}{{\,\mathrm{{\mathcal {V}}}\,}}_c(f_k,x)\bigr ) < 8\pi &{} \text {if }c_0\ge 0. \end{array}\right. } \end{aligned}$$

Then the infimum is attained by a smooth embedding $f:{\mathbb {S}}^2\rightarrow {\mathbb {R}}^3$.

#### Remark 6.11

(i) In view of (6.15), (6.18) and Lemma 6.4, we see that if $c_0\le 0$, then
  $$\begin{aligned} \inf _{x_0\in f[{\mathbb {S}}^2]} {{\,\mathrm{{\mathcal {V}}}\,}}_c(f,x_0) \ge \frac{(2\pi )^2{{\,\mathrm{{\mathcal {V}}}\,}}(f)}{9^2({\mathcal {A}}(f) + \frac{2}{3}|c_0|{{\,\mathrm{{\mathcal {V}}}\,}}(f))}\frac{1}{{\mathcal {H}}_{c_0}(f)} \end{aligned}$$
  for all $f\in {\mathcal {Q}}$. Thus, an elementary computation shows that (6.40) is satisfied provided
  $$\begin{aligned} {\bar{\eta }}(c_0,A_0,V_0) < 4\pi \left( 1+ {\sqrt{1 + L(c_0,A_0,V_0)}}\right) \end{aligned}$$
  for
  $$\begin{aligned} L(c_0,A_0,V_0):=\frac{|c_0|V_0}{2\cdot 9^2(A_0 + \frac{2}{3}|c_0|V_0)} > 0. \end{aligned}$$
(ii) Using (6.16), (5.2) and (6.18), for all $r>0$ and $f\in {\mathcal {Q}}$ we have
  $$\begin{aligned} \sup _{x_0\in f[{\mathbb {S}}^2]}{{\,\mathrm{{\mathcal {V}}}\,}}_c(f,x_0)=\sup _{x_0\in f[{\mathbb {S}}^2]}\int \limits _{E}|x-x_0|^{-2}\mathop {}\!\textrm{d}{\mathcal {L}}^3(x)\le 4\pi r + r^{-2}{{\,\mathrm{{\mathcal {V}}}\,}}(f). \end{aligned}$$
  Minimizing over $r>0$ yields the estimate ${{\,\mathrm{{\mathcal {V}}}\,}}_c(f,x_0) \le 3(4\pi ^2 {{\,\mathrm{{\mathcal {V}}}\,}}(f))^{\frac{1}{3}}$. Thus, (6.40) is satisfied for $c_0>0$ provided
  $$\begin{aligned} {\bar{\eta }}(c_0,A_0,V_0) < 8\pi - 6c_0(4\pi ^2 V_0)^{\frac{1}{3}}. \end{aligned}$$
(iii) For all $c_0\le 0$ and $\sigma \ge 36\pi$, there exists ${\bar{A}}_0, {\bar{V}}_0>0$ such that ${\bar{A}}_0^3/{\bar{V}}_0^2=\sigma$ and ${\bar{\eta }}(c_0,A_0,V_0) <8\pi$ for all $0< A_0<{\bar{A}}_0$, $0<V_0<{\bar{V}}_0$ with $A_0^3/V_0^2=\sigma$. Indeed, in view of (1.2), this is a consequence of [41, Lemma 1].

#### Proof of Theorem 6.10

By (6.27), we have that

$$\begin{aligned} \sup _{k\in {\mathbb {N}}} {\mathcal {W}}(f_k) \le C(c_0,A_0,V_0) < \infty . \end{aligned}$$

Hence, by Lemma 6.3 applied for $c_0 = 0$, there holds $\inf _{k\in {\mathbb {N}}} {{\,\textrm{diam}\,}}f_k[{\mathbb {S}}^2] > 0$. Moreover, after translations, we may assume $0\in f_k[{\mathbb {S}}^2]$ for all *k*. Therefore, we can apply Lemma 6.8 to obtain $f\in {\mathcal {Q}}$ injective such that, after passing to a subsequence,

$$\begin{aligned} V_k \rightarrow V \qquad \text {in }{\mathbb {V}}_2^\textrm{o}({\mathbb {R}}^3)\text { as }k\rightarrow \infty , \end{aligned}$$

where $V_k,V$ are the oriented 2-varifolds in ${\mathbb {R}}^3$ associated with $f_k,f$. The varifold convergence implies ${\mathcal {A}}(f) = A_0$ and ${{\,\mathrm{{\mathcal {V}}}\,}}(f) = V_0$. Thus, by (6.25), *f* attains the infimum (6.39).

Let $\omega \in C^\infty ({\mathbb {S}}^2,{\mathbb {R}}^3)$ and define $f_t :=f + t\omega$ for $t\in {\mathbb {R}}$. By (6.9) and (6.13) we have

$$\begin{aligned}&f_t \rightarrow f{} & {} \text {in }W^{1,\infty }({\mathbb {S}}^2;{\mathbb {R}}^3)\cap W^{2,2}({\mathbb {S}}^2;{\mathbb {R}}^3)\text { as }t\rightarrow 0,\\&\mathrm df_t\wedge \mathrm df_t \rightarrow \mathrm df \wedge \mathrm df{} & {} \text {in }L^\infty ({\mathbb {S}}^2;({\textstyle \bigwedge }_2T^*{\mathbb {S}}^2)\otimes {\textstyle \bigwedge }_2{\mathbb {R}}^3)\text { as }t\rightarrow 0,\\&n_t\rightarrow n{} & {} \text {in }L^\infty ({\mathbb {S}}^2;{\mathbb {R}}^3)\text { as }t\rightarrow 0 \end{aligned}$$

and the associated varifolds converge in $\mathbb V_2^\textrm{o}({\mathbb {R}}^3)$. Moreover, it follows that $f_t\in {\mathcal {E}}$ for |*t*| small and ${\mathcal {W}}(f_t) \rightarrow {\mathcal {W}}(f)$, ${\mathcal {H}}_{c_0}(f_t) \rightarrow {\mathcal {H}}_{c_0}(f)$, and ${\mathcal {A}}(f_t) \rightarrow {\mathcal {A}}(f)$ as $t\rightarrow 0$. Hence, similarly as in (6.37) and (6.38), we can combine Lemma 3.6 and Lemma 3.7 to deduce that for some $\varepsilon >0$ there holds

$$\begin{aligned} {\mathcal {H}}_{c_0}(f_t) + 2 c_0{{\,\mathrm{{\mathcal {V}}}\,}}_c(f_t,x_0)< 8\pi \qquad \text {for all }|t|<\varepsilon \text { and }x_0\in f_t[{\mathbb {S}}^2]. \end{aligned}$$

It follows by Corollary 4.4 that $f_t$ is injective for $|t|<\varepsilon$ and thus, by Lemma 6.6 and Remark 6.7, $f_t\in {\mathcal {Q}}$. Therefore, we can proceed as in [31] and [33] to deduce that *f* satisfies the Euler–Lagrange equation given in [31, Lemma 4.1]. Now, we can apply [31, Theorem 4.3] to conclude that *f* is smooth. $\square$

Theorem 1.6 is now a direct consequence.

#### Proof of Theorem 1.6

We will prove the theorem in the case where

$$\begin{aligned} \Gamma (c_0, A_0, V_0) = {\left\{ \begin{array}{ll} 4\pi \left( \sqrt{1+L(c_0, A_0, V_0)}-1\right) &{} \text { if }c_0< 0, \\ -6 c_0 \left( 4\pi ^2 V_0\right) ^{\frac{1}{3}} &{}\text { if }c_0\ge 0, \end{array}\right. } \end{aligned}$$

with $L(c_0, A_0, V_0)$ as in Remark 6.11(i).

Let $f_k \in {\mathcal {Q}}$ be a minimizing sequence for (6.39). By Remark 6.11(i) and (ii), the choice of $\Gamma$, and since ${\bar{\eta }}(c_0, A_0, V_0)\le \eta (c_0, A_0, V_0)$ as a consequence of Lemma 6.6, we find that (6.40) is satisfied and hence the infimum (6.39) is attained by a smooth embedding $f:{\mathbb {S}}^2\rightarrow {\mathbb {R}}^3$, which implies that *f* is also a minimizer for (1.9) and thus ${\bar{\eta }}(c_0, A_0, V_0)=\eta (c_0, A_0, V_0)$. The last part of Theorem 1.6 follows from Remark 6.11(iii). $\square$

### Positive total mean curvature

We recall the following inequality due to Minkowski [29]. If $\Omega \subset {\mathbb {R}}^3$ is a bounded convex open subset with $C^2$-boundary $\partial \Omega$, then

6.41 $$\begin{aligned} \frac{1}{2}\int \limits _{\partial \Omega } H_{\textrm{sc}} \mathop {}\!\textrm{d}{\mathcal {H}}^2\ge \sqrt{4\pi {\mathcal {H}}^2(\partial \Omega )}, \end{aligned}$$

with equality if and only if $\Omega$ is a ball. The quantity on the left hand side of (6.41) is called *total (scalar) mean curvature*. With the help of Corollary 5.3, we can generalize (6.41) to a class of nonconvex surfaces.

#### Theorem 6.12

Let $f:\Sigma \rightarrow {\mathbb {R}}^3$ be an immersion of an oriented closed surface $\Sigma$. If there exists $x_0\in {\mathbb {R}}^3$ with ${{\,\mathrm{{\mathcal {V}}}\,}}_c(f,x_0)>0$ and $\bar{{\mathcal {H}}}(f) \le 4\pi {\mathcal {H}}^{0}(f^{-1}\{x_0\})$, then we have

6.42 $$\begin{aligned} \frac{1}{2}\int H_{\textrm{sc}}\mathop {}\!\textrm{d}\mu \ge \sqrt{\left( 4\pi {\mathcal {H}}^{0}(f^{-1}\{x_0\})-\bar{{\mathcal {H}}}(f)\right) {\mathcal {A}}(f)}. \end{aligned}$$

The assumption ${{\,\mathrm{{\mathcal {V}}}\,}}_c(f,x_0)>0$ is especially satisfied if *f* is an Alexandrov immersion and $x_0\in {\mathbb {R}}^3$ is arbitrary, see (5.1).

We would like to point out that it is possible to deduce (6.42) with the absolute value on the left hand side from the classical Li–Yau inequality for the Willmore energy. However, the question whether the total mean curvature is positive remains. In general, this has to be answered in the negative; however, under certain convexity or symmetry assumptions on the surface, the total mean curvature can be shown to be positive, cf. [12, Table 1]. In the case of Alexandrov immersions, Theorem 6.12 provides a sufficient criterion for positive total mean curvature if the CMC-deficit is not too large, depending on the concentrated volume and the multiplicity at a point.

#### Proof of Theorem 6.12

Set $\delta :=4\pi {\mathcal {H}}^{0}(f^{-1}\{x_0\})-\bar{{\mathcal {H}}}(f)\ge 0$. By Corollary 5.3 we have

$$\begin{aligned} \delta {\mathcal {A}}(f) \le {2\int \limits _{\Sigma } H_{\textrm{sc}}\mathop {}\!\textrm{d}\mu ~ {{\,\mathrm{{\mathcal {V}}}\,}}_{c}(f,x_0)} - 4{{\,\mathrm{{\mathcal {V}}}\,}}_{c}(f, x_0)^2, \end{aligned}$$

and therefore, using Young’s inequality and ${{\,\mathrm{{\mathcal {V}}}\,}}_c(f,x_0)>0$, we find

$$\begin{aligned} \frac{\int H_{\textrm{sc}}\mathop {}\!\textrm{d}\mu }{2} \ge \frac{\delta {\mathcal {A}}(f)}{4 {{\,\mathrm{{\mathcal {V}}}\,}}_c(f, x_0)} +{{\,\mathrm{{\mathcal {V}}}\,}}_c(f,x_0) \ge \sqrt{\delta {\mathcal {A}}(f)}. \square \end{aligned}$$
